# The association between Zika virus infection and microcephaly in Brazil 2015–2017: An observational analysis of over 4 million births

**DOI:** 10.1371/journal.pmed.1002755

**Published:** 2019-03-05

**Authors:** Oliver J. Brady, Aaron Osgood-Zimmerman, Nicholas J. Kassebaum, Sarah E. Ray, Valdelaine E. M. de Araújo, Aglaêr A. da Nóbrega, Livia C. V. Frutuoso, Roberto C. R. Lecca, Antony Stevens, Bruno Zoca de Oliveira, José M. de Lima, Isaac I. Bogoch, Philippe Mayaud, Thomas Jaenisch, Ali H. Mokdad, Christopher J. L. Murray, Simon I. Hay, Robert C. Reiner, Fatima Marinho

**Affiliations:** 1 Department of Infectious Disease Epidemiology, London School of Hygiene & Tropical Medicine, London, United Kingdom; 2 Institute for Health Metrics and Evaluation, University of Washington, Seattle, Washington, United States of America; 3 Department of Anesthesiology & Pain Medicine, University of Washington, Seattle, Washington, United States of America; 4 Secretariat of Health Surveillance, Ministry of Health of Brazil, Brasília, Brazil; 5 Division of Infectious Diseases, Department of Medicine, University of Toronto, Toronto, Ontario, Canada; 6 Division of General Internal Medicine, University Health Network, Toronto, Ontario, Canada; 7 Division of Infectious Diseases, University Health Network, Toronto, Ontario, Canada; 8 Department of Clinical Research, London School of Hygiene & Tropical Medicine, London, United Kingdom; 9 Section of Clinical Tropical Medicine, Department of Infectious Diseases, Heidelberg University Hospital, Heidelberg, Germany; University of Manchester, UNITED KINGDOM

## Abstract

**Background:**

In 2015, high rates of microcephaly were reported in Northeast Brazil following the first South American Zika virus (ZIKV) outbreak. Reported microcephaly rates in other Zika-affected areas were significantly lower, suggesting alternate causes or the involvement of arboviral cofactors in exacerbating microcephaly rates.

**Methods and findings:**

We merged data from multiple national reporting databases in Brazil to estimate exposure to 9 known or hypothesized causes of microcephaly for every pregnancy nationwide since the beginning of the ZIKV outbreak; this generated between 3.6 and 5.4 million cases (depending on analysis) over the time period 1 January 2015–23 May 2017. The association between ZIKV and microcephaly was statistically tested against models with alternative causes or with effect modifiers. We found no evidence for alternative non-ZIKV causes of the 2015–2017 microcephaly outbreak, nor that concurrent exposure to arbovirus infection or vaccination modified risk. We estimate an absolute risk of microcephaly of 40.8 (95% CI 34.2–49.3) per 10,000 births and a relative risk of 16.8 (95% CI 3.2–369.1) given ZIKV infection in the first or second trimester of pregnancy; however, because ZIKV infection rates were highly variable, most pregnant women in Brazil during the ZIKV outbreak will have been subject to lower risk levels. Statistically significant associations of ZIKV with other birth defects were also detected, but at lower relative risks than that of microcephaly (relative risk < 1.5). Our analysis was limited by missing data prior to the establishment of nationwide ZIKV surveillance, and its findings may be affected by unmeasured confounding causes of microcephaly not available in routinely collected surveillance data.

**Conclusions:**

This study strengthens the evidence that congenital ZIKV infection, particularly in the first 2 trimesters of pregnancy, is associated with microcephaly and less frequently with other birth defects. The finding of no alternative causes for geographic differences in microcephaly rate leads us to hypothesize that the Northeast region was disproportionately affected by this Zika outbreak, with 94% of an estimated 8.5 million total cases occurring in this region, suggesting a need for seroprevalence surveys to determine the underlying reason.

## Abstract 2

Oliver Brady and colleagues reveal an association between congenital ZIKA infection during the first 2 trimesters of pregnancy and microcephaly using data from a large Brazilian cohort.

## Author summary

**Why was this study done?:**

- In late 2015 above average rates of the congenital malformation microcephaly were reported in Northeast Brazil.
- This coincided with the first introduction of Zika in the Americas, and evidence of the association between Zika virus infection in pregnancy and microcephaly shortly followed.
- However, considerably lower rates of microcephaly were later observed elsewhere in Brazil and in other areas to which Zika subsequently spread.
- This led to suggestions of alternative causes or cofactors present in Northeast Brazil and uncertainty over the magnitude of risk that women infected with Zika in pregnancy faced.

**What did the researchers do and find?:**

- In this study we attempted to assemble information on 9 known or hypothesized causes of microcephaly for every birth in Brazil since the beginning of the Zika outbreak. We then statistically tested different hypotheses about the cause of the 2015–2017 microcephaly outbreak nationwide.
- We found no evidence for alternative non-Zika causes of the microcephaly outbreak nor did we find evidence that microcephaly risk was modified by concurrent dengue infection or previous yellow fever vaccination.
- Women infected with Zika virus early in pregnancy were 17 times (95% confidence interval 3–369) more likely to have a child with microcephaly.
- We found that microcephaly risk was highest in women with Zika infection in the first 2 trimesters, and that these women may also be at higher risk of developing other non-microcephaly birth defects, but that this is a rarer outcome of congenital Zika infection.

**What do these findings mean?:**

- These findings add to the growing evidence that Zika infection early in pregnancy has a generalisable association with microcephaly and, to a lesser extent, a range of other birth defects.
- What this study adds is the ability to map the impact of Zika across broad areas, and it leads us to hypothesize that Northeast Brazil experienced a significantly more substantial Zika outbreak than the rest of the country, with 92% of an estimated 8.5 million Zika cases.
- Community-representative seroprevalence surveys across Brazil are needed to test this hypothesis and give insights into the risk of further Zika outbreaks in Brazil.

## Introduction

Near the end of 2015, an abnormally high rate of the congenital malformation microcephaly was reported from the 7 of the states in Northeast Brazil [1]. This event coincided with the first known introduction of the Zika virus (ZIKV) into South America, which subsequently spread throughout the continent, causing the World Health Organization to declare a Public Health Emergency of International Concern in February 2016 [2].

Since then, a growing body of evidence has linked ZIKV infection during pregnancy with microcephaly and other adverse birth outcomes [3–6]. There are also suggestions that ZIKV could be associated with a wider range of congenital and developmental abnormalities, some of which may not be apparent until later in a child’s life [4,7,8]. The rates of microcephaly initially reported in states in Northeast Brazil (up to 56.7 per 10,000 births) have not been documented in other Brazilian states (5.5–14.5 per 10,000 births) nor in other countries to which ZIKV has since spread [9–11].

Microcephaly is a manifestation of in utero growth abnormalities that lead to an abnormally small head size. In more severe cases, growth restriction affects the brain and central nervous system (CNS), which may lead to considerable permanent cognitive impairments, if the infant survives. Microcephaly is a known outcome of a variety of factors, including chromosomal disorders and other genetic syndromes, infections during pregnancy (e.g., rubella, toxoplasmosis), and exposure to harmful toxins during pregnancy. In the absence of major viral outbreaks, the condition affects between 0.4 and 4.3 individuals per 10,000 births [12]. Measuring this baseline rate in Brazil has been challenging due to changing definitions of the condition [11] and considerable subnational variation. In Brazil at the time of the Zika epidemic, a diagnosis of ‘suspected microcephaly’ was given if the infant’s head circumference was significantly below average at birth (<2 standard deviations below the mean for gestational age and sex). Presence of CNS growth defects can only be confirmed through cranial imaging techniques, typically via computerised tomography (CT) or magnetic resonance imaging (MRI) scans. Children with a small head circumference at birth and with imaging evidence of abnormal brain or CNS development were given a definition of ‘confirmed microcephaly’ in routine reporting systems. Limited availability of diagnostic imaging in Brazil in late 2015 meant many suspected cases were still under investigation, thus hampering the interpretation of reported rates at the time. Since then, follow-up testing of affected children has addressed some of these gaps in surveillance for microcephaly.

Geographic differences in microcephaly rates since the ZIKV outbreak began have prompted suggestions that there may be additional, as-of-yet unknown effect modifiers or causes at play in Northeast Brazil [13]. These could include coinfection with, or immune enhancement following past exposure to, flaviviruses and alphaviruses that are closely related to ZIKV such as dengue and chikungunya viruses. Enhancement of Zika disease following prior dengue virus exposure has been demonstrated in vitro [14] and in mice models [15] but has been questioned in analyses of human sera [16]. The rural distribution of reported microcephaly cases in some areas (an unusual feature given the urban preference of ZIKV-transmitting *Aedes* mosquitoes) has also led some to suggest the possible involvement of drinking water contamination [13] or zoonotic causes such as bovine viral diarrhoea virus (BVDV), which can cause birth defects in cattle [17].

Testing such hypotheses has been challenging for a combination of practical and theoretical reasons. First, sample sizes for prospective cohort studies would need to be prohibitively large to test multiple effect modifier hypotheses for a disease where up to 65%–83% of cases may be asymptomatic [18,19] and at a time when the epidemic is in decline. Second, retrospective analyses of routine surveillance data need to account for changing definitions of microcephaly and changing practices in Zika case notification over time and be at a sufficiently fine geographic scale to relate one to another. Third, there remains considerable uncertainty in the baseline rate of non-ZIKV-attributable microcephaly and its subnational variation.

In this study, we tested for an association between ZIKV and a range of congenital malformations while controlling for other causes that may have influenced the increase in microcephaly observed in Brazil. We used multiple national-level databases to estimate the level of ecological exposure to ZIKV and other known and hypothesized causes of microcephaly for each pregnancy since the beginning of the ZIKV outbreak. We then used this large dataset to statistically compare different hypotheses for the observed pattern of microcephaly, with the aim of characterising the magnitude and statistical significance of the ZIKV–microcephaly association.

## Methods

### Data sources

For every live birth in Brazil, the presence or absence of congenital abnormalities is recorded in national health statistics. Information on exposure of each of these pregnancies to a range of harmful pathogens, such as ZIKV, is usually not obtained or recorded, however. As a result, our approach relied on producing an individually tailored high-resolution estimate of likely exposure over pregnancy by matching pregnancy timing (week between conception and birth) and location (municipality of residence of the mother) to information on candidate exposures (Zika, dengue, chikungunya, BVDV, and water toxins) contained in other databases. Given the brief duration of the ZIKV outbreak in most municipalities (mean of 3 weeks; S1 Text, section 1.1), this approach provides a much more specific measure of exposure than aggregated data.

Individual-level data on all live births between 1 January 2015 and 23 May 2017 were extracted from the Sistema de Informações sobre Nascidos Vivos (SINASC) database, which also included data on age and race of the mother, sex of the baby, pregnancy date, and municipality of residence of the mother (5,485 municipalities). Any birth defects apparent at birth were recorded in SINASC using ICD-10 standards. Since the beginning of December 2015, notification of microcephaly to the Registro de Eventos em Saúde Pública (RESP) system—manifest in utero, at birth, or postpartum—has been mandatory. Here we distinguish 2 types of microcephaly: morphological microcephaly (MM) and microcephaly with structural CNS defects (hereafter termed microcephaly with structural brain defects [MWSD]), originally reported as ‘suspected microcephaly’ and ‘confirmed microcephaly’, respectively.

From 13 March 2016 onwards [20,21], MM was diagnosed if the infant’s head circumference at birth was less than or equal to 31.9 cm for male or 31.5 cm for female babies. MWSD was diagnosed if subsequent imaging techniques (primarily CT and MRI scanning; S1 Text, section 1.2) revealed brain or CNS developmental abnormalities. Here we focus our analysis on births with MWSD, as MWSD is the more severe outcome of the condition and is less affected by natural variations in infant growth size. We also performed the same analyses on MM and report all results in S2 Text, section 2.4. Records with MWSD in RESP (cases) were standardised to the definition used from March 2016 onwards, then matched to their corresponding record in SINASC using a deterministic matching algorithm (S1 Text, section 1.6), with identified duplicates removed from SINASC. All MWSD cases (RESP) were then compared to all control (non-microcephaly) births (SINASC).

**Fig 1:**
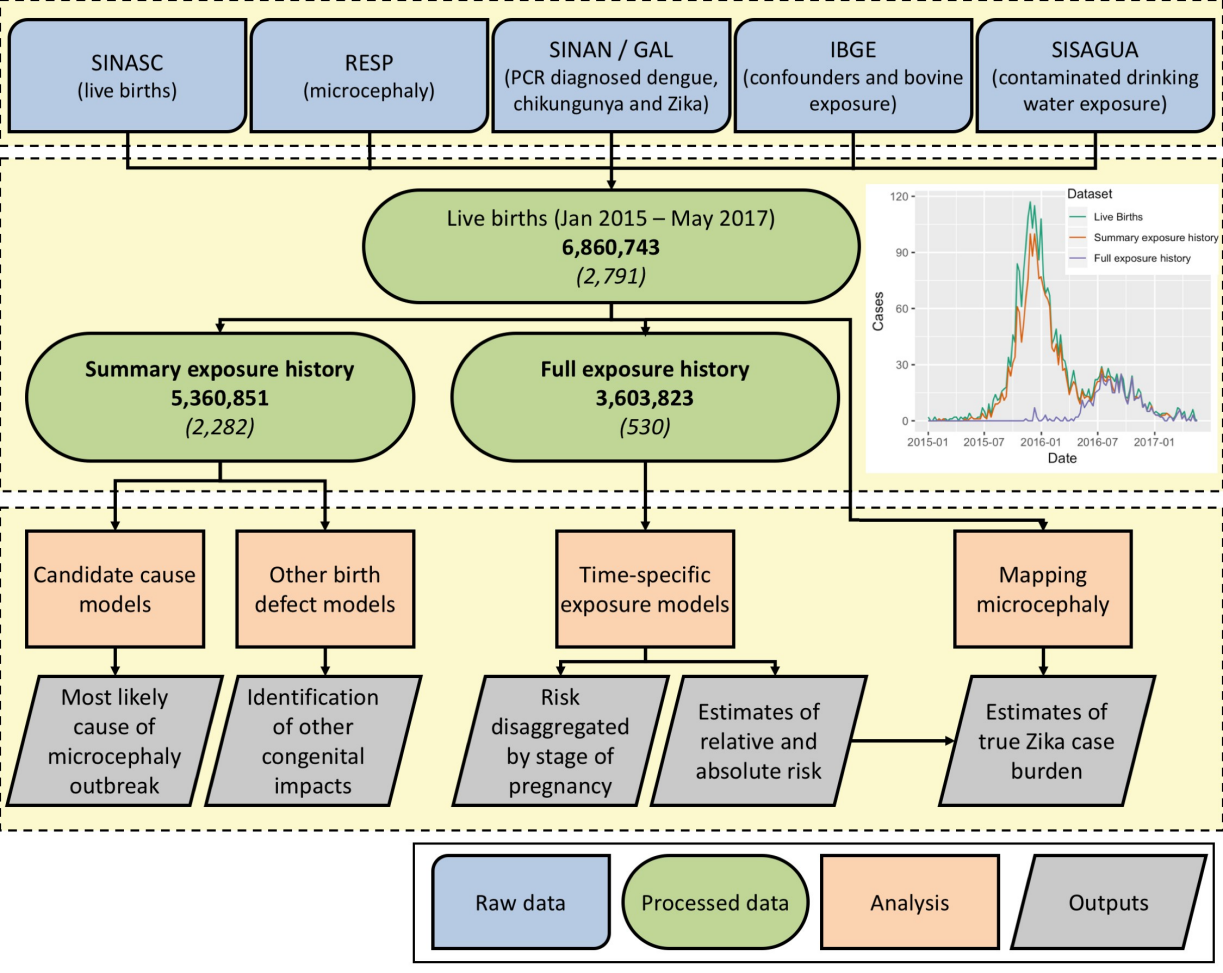
Data sources, data processing, and analysis schema. Numbers in bold indicate total number of births in each category; numbers in italics indicate number of cases of microcephaly with structural brain defects (MWSD) in each category. IBGE, Instituto Brasileiro de Geografia e Estatística; RESP, Registro de Eventos em Saúde Pública; SINAN/GAL, Sistema de Informação de Agravos de Notificação/Gerenciador de Ambiente Laboratorial; SINASC, Sistema de Informações sobre Nascidos Vivos; SISAGUA, Sistema de Informação de Vigilância da Qualidade da Água para Consumo Humano.

Weekly Zika, dengue, and chikungunya PCR-confirmed cases in each municipality were obtained from the Sistema de Informação de Agravos de Notificação (SINAN). We only considered Zika data as reliable after the date of 8 December 2015; before this date Zika reporting was sporadic, non-compulsory, and not PCR tested, and therefore we assign ‘no data’ values to all weeks before this date. Weekly counts were converted into incidence rates using municipality-level population data to give a probability of infection within each municipality for each week [22]. For each pregnancy, the mean weekly incidence experienced between conception and the week of birth (candidate cause and other birth defect models; Fig 1) and the mean weekly incidence among defined stages of pregnancy (time-specific exposure models) were calculated. Weeks with missing data were omitted from the mean calculation (i.e., we assumed these data were missing at random). We chose to focus our analysis on PCR-confirmed cases due to a lack of specificity of clinical diagnosis among arboviral diseases with similar symptoms. As much of the PCR testing was done at central laboratories, little geographic variation in testing rate or positivity rate was observed (S1 Text, section 1.1). We also repeated the analysis using clinically suspected cases of Zika, dengue, and chikungunya and report the results in S2 Text, section 2.4.

Yellow fever vaccination coverage in each municipality in the year 2016 was obtained from Shearer et al. [23] (untargeted unbiased scenario), which used multi-year data on age-specific doses delivered from the Brazilian national immunisation programme. National data on incidence of waterborne pesticides and toxins were not available. Instead we used a proxy of exposure, a time-varying drinking water quality index (S1 Text, section 1.3). For exposure to potential bovine viruses, viral agent testing data were unavailable, so we used the density of farms per square kilometre in each municipality as a proxy for exposure.

The socio-demographic status of each municipality was quantified using a socio-demographic index (S1 Text, section 1.5) that distinguishes levels of income, education, and fertility between areas using data from the 2010 Brazilian census [22].

All disease incidence rates, yellow fever vaccination rates, and cattle density variables were zero-inflated by half the minimum observed incidence value then log transformed. No collinearity was detected between all combinations of covariates (all variance inflation factors below 2.5).

### Data selection

Missing or implausible information at the individual level was found in a small number of records (0.28%; S1 Text, section 1.4), which were removed from the regression analysis (assumed missing at random, minimal difference in characteristics; S1 Text, section 1.4).

Because ZIKV surveillance was only established nationwide in December 2015, and the majority of MWSD cases occurred in early 2016, we were not able to identify full exposure histories for the majority of MWSD cases. We therefore generated 2 separate datasets: (i) a ‘summary exposure history’ dataset that requires information on exposure to ZIKV during 1 or more weeks of pregnancy and (ii) a ‘full exposure history’ dataset that requires information on exposure to ZIKV in at least 1 week in each of trimesters 1, 2, and 3 as well as the time period 10 weeks prior to conception (Fig 1 and S1 Text, section 1.4). These 2 data selections balance minimising data selection (and thus selection bias and maintaining sample size) and accurately defining exposure.

### Hypothesis testing for causes of MWSD

To identify the most likely cause of the excess MWSD cases observed in Brazil, a number of candidate statistical models with different hypothesized exposures were formally compared. Candidate primary exposures during pregnancy included (i) Zika, (ii) dengue, (iii) chikungunya, (iv) BVDV, and (v) water toxins. In addition, we also tested for interaction exposures between Zika and (i) dengue, (ii) chikungunya, and (iii) prior yellow fever vaccination. Interaction exposures are intended to test if coinfection (or sequential infection within pregnancy) confers risk beyond ZIKV exposure alone or if prior yellow fever vaccine elevates risk for exposures to ZIKV in pregnancy. Interaction exposures were calculated by multiplying the ZIKV exposure variable by the candidate interaction exposures. Due to the rarity of some congenital abnormalities, including MWSD, Firth’s penalised logistic regression [24] was chosen to test for associations in all analyses. The summary exposure history dataset was chosen for this analysis because of its high representativeness of the early stages of the outbreak; however, this does mean that the estimates of relative risk may be unreliable (biased towards the null) if exposure in early pregnancy has a greater effect than exposure at the end (see timing of exposure analysis).

Because MWSD can also be caused by numerous other causes other than our candidate exposures (e.g., environmental causes, genetic causes, and toxoplasmosis, other, rubella, cytomegalovirus, herpes, and syphilis [TORCH(S)] infections), in all exposure models we tested for the inclusion of the following baseline risk factor variables: (i) mother’s age, (ii) sex of the baby, and (iii) socioeconomic status. These variables have all previously been found to be associated with a range of birth defects [25–27]. We also included variables for week (beginning 1 January 2015) and region (5 regions) to account for spatio-temporal variance not explained by any of the other covariates.

All combinations of baseline risk factors were tested in combination with each hypothesized exposure (32 baseline risk factor combinations × 8 hypothesized exposures = 256 candidate models). The optimal combination of baseline risk factors for each exposure was chosen by minimum Akaike Information Criterion (AIC), which was also used to compare between exposures to identify the most parsimonious explanation for the observed pattern of MWSD.

### Testing for associations between ZIKV and non-microcephaly birth defects

To test for an association between ZIKV and multiple non-microcephaly birth defects while maintaining family-wide error rates, we prioritised hypothesis testing of (i) groups of birth defects suspected or previously linked to maternal ZIKV infection and (ii) high-frequency (at least 2 per 10,000 births) specific birth defects (S2 Text, section 2.6). For each birth defect outcome, optimal baseline risk factor variables were selected by minimum AIC to account for non-ZIKV causes of each birth defect. This analysis also used the summary exposure history dataset to preserve sample size and because we had no prior suggestion that exposure late in pregnancy differed from early exposure. Because of the large number of outcomes tested for association (*n* = 12), Bonferroni correction was used to adjust *p*-values to maintain *p* = 0.05 equivalence.

### Assessing timing of risk during pregnancy

To identify in which gestational periods maternal ZIKV infection was associated with increased MWSD risk, we repeated the regression analysis with exposure disaggregated into 1 of 4 categories: prior to conception (up to 10 weeks prior), trimester 1, trimester 2, and trimester 3. This required using the full exposure history dataset that excluded many of the cases from the first phase of the epidemic but had more complete information on each mother’s exposure over the duration of her pregnancy. Baseline risk factors were included and selected by minimum AIC; however, the single continuous covariate for ‘week’ and the 5-category ‘region’ covariate were further disaggregated to better account for residual spatial and temporal correlation and thus provide unbiased estimates of ZIKV relative risk. A fixed effect term for each month between October 2015 and July 2016 (coinciding with the main MWSD outbreak) as well as terms for the years 2015, 2016, and 2017 for months outside this range were included (increase from 1 to 12 temporal effects). Additionally, all births in states with incidence greater than 9 MWSD cases per 10,000 live births were assigned a state-level fixed effect (increase from 5 to 12 spatial effects).

The cumulative ZIKV incidences in trimesters 1 and 2 were found to be partially colinear (*R*^2^ = 0.75) and were combined into a single covariate (cumulative Zika incidence in the first 2 trimesters). Further analysis with separated trimester data (detailed in S2 Text, section 2.5) investigated the possible differential effects between exposure in trimesters 1 and 2.

### Mapping MWSD relative and absolute risks and estimating Zika case burden

Because microcephaly is a rare pregnancy outcome, a large number of births are required to give accurate crude estimates of the true rate of microcephaly in each municipality. Because such large numbers of births did not occur in many municipalities (S1 Text, section 1.2), we generated smooth surfaces of microcephaly rate (averaged over the period January 2015–May 2017) that accounted for variable birth counts using a generalised additive model (GAM).

The GAM included counts of births with and without MWSD as the response and (i) a 2-dimensional thin-plate spline of latitude and longitude, (ii) the municipality-wide mean predicted ZIKV probability of occurrence from a Zika ecological niche map [28], (iii) the mean socio-demographic index level of mothers in each municipality, and (iv) the mean age of mothers in each municipality. Thin-plate splines were chosen for their 2-dimensional support, with smoothing parameters selected by generalised cross validation with high starting optimisation values (*k* = 1,000) to capture high spatial heterogeneity.

To estimate Zika case burden over the course of the entire epidemic, we back-projected the incidence of Zika needed to generate the rate of MWSD cases observed according to our best estimates of the ZIKV–MWSD association. We used the results from the time-specific exposure model (Fig 1); while it was fit mostly to records in the second wave of the epidemic (Fig 1), it contained the most precise definition of exposure and generated the best estimate of absolute and relative risk. For each municipality, all births over the time period were collated, and the regression model coefficients (mean and 95% confidence intervals) were used to predict the baseline (i.e., in the absence of Zika) expected rate of MWSD cases (Jan 2015–May 2017). The residual between the predicted and observed rate of MWSD cases (after smoothing with the mean prediction from the GAM mapping analysis) was combined with the parameter estimates from the regression equation and used to solve for the ZIKV incidence needed in each pregnancy to generate the observed MWSD rates.

All analyses were conducted in R version 3.3.3 [29] using the ‘logistf’ and ‘mgcv’ packages. While we are not able to publicly share the individual-level data on pregnancies and their health outcomes used in this analysis due to identifiability concerns, summaries aggregated at a high spatio-temporal resolution (municipality and week) are freely available from the following online repository: https://doi.org/10.6084/m9.figshare.7359197.v1. We have made the code used to analyse the data publicly available and have provided extensive summaries of the dataset in S1 Text, section 1.1 and 1.2, to increase transparency (https://github.com/obrady/Brazil_microcepahly_analysis_public). This study was approved by the London School of Hygiene & Tropical Medicine ethics committee (Ref: 14603). The full institutional review board (IRB) submission can be found in S1 IRB, the statistical analysis plan in S1 Analysis Plan, and the STROBE checklist in S1 STROBE. Subsequent deviations from the analysis plan are detailed in S1 Text, section 1.9, which included the need to define separate summary and full exposure datasets due to the late establishment of Zika surveillance. Initial and modified results and a discussion of the relevant changes are given in S1 Text, section 1.9.

## Results

A total of 6,860,743 births occurred between 1 January 2015 and 23 May 2017. A total of 2,791 of these births resulted in MWSD, with the largest concentration between November 2015 and May 2016 and elevated levels continuing to be observed throughout the rest of 2016 (Fig 1). Because Zika surveillance was sporadic before December 2015, we selected 2 separate datasets: summary exposure history (information on exposure at some point in pregnancy) and full exposure history (information on exposure throughout pregnancy). In the summary exposure dataset 2,202,714 (41%) pregnancies were potentially exposed to ZIKV at varying levels.

### Cause of the 2015–2017 microcephaly outbreak

First, we tested for the presence of effect modifiers and alternative causes most likely to explain the microcephaly epidemic observed in Brazil. This analysis was performed on the summary exposure history dataset (Fig 1; 5,360,851 pregnancies with 2,282 MWSD cases). This maintained representativeness of the early stages of the outbreak but may bias estimates of relative risk towards the null.

**Fig 2:**
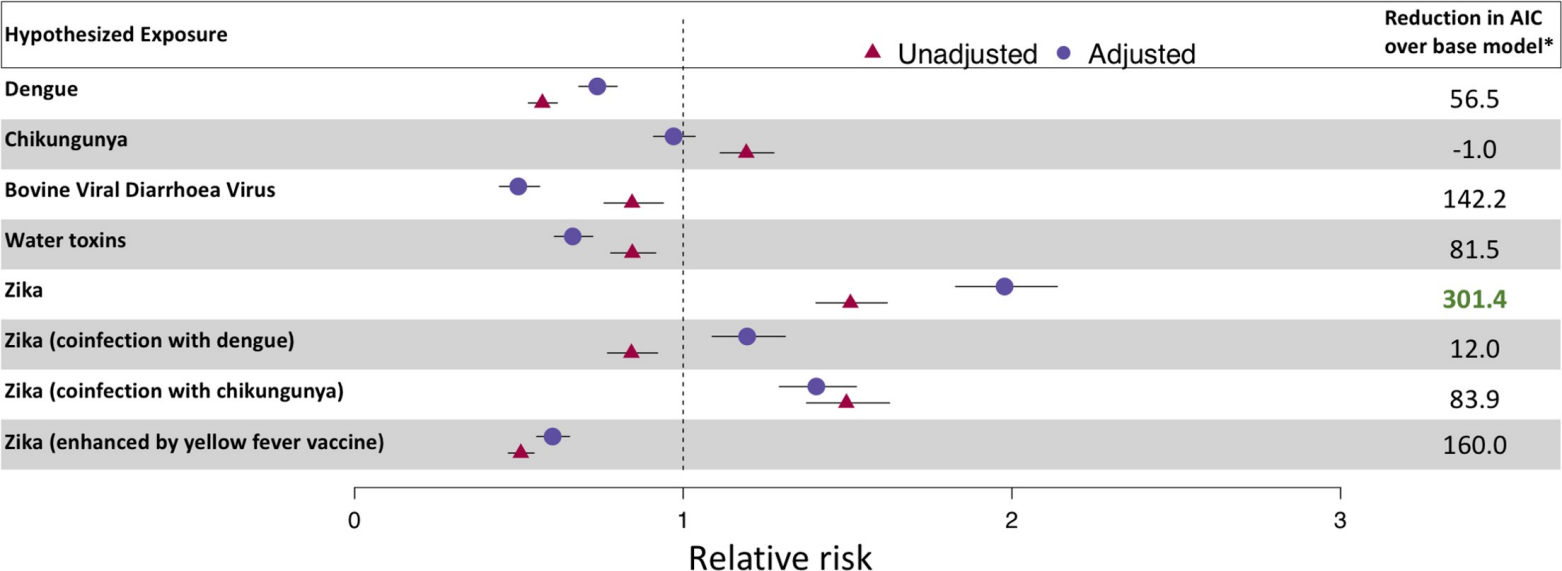
Association between candidate exposures and microcephaly with structural brain defects. Relative risk compares areas with no exposure to those with median incidence of the exposure. Adjusted models included variables for region and sex of the baby (only bovine viral diarrhoea virus, Zika, and Zika [chikungunya coinfection] models; S2 Text, section 2.9). *The final column shows the Akaike Information Criterion (AIC) of a base model including a covariate for region minus the AIC of the adjusted exposure model (a larger reduction in AIC value equates to a more likely exposure model).

To distinguish the causes of this microcephaly epidemic from the long-term average background rate of MWSD and its spatial variation, we included a range of background risk variables in each regression. In nearly all models the covariates of sex of the baby and region were retained (higher risk in the Northeast region and in female babies) while mother’s age, week, and socio-demographic index level were removed by model selection (Fig 2).

Fig 2 shows the relative risks of MWSD given exposure to a range of candidate causes and effect modifiers before and after adjusting for the local background rate. Exposure to ZIKV alone is the most likely explanation of the observed pattern of MWSD, showing a significantly larger reduction in AIC value over the baseline (no exposure) model than other hypothesized exposures or models that considered arboviral effect modifiers (Fig 2). While other exposures do show smaller magnitude but statistically significant associations with MWSD (e.g., dengue and chikungunya in Fig 2), these were likely due to partial collinearity with Zika transmission as they did not explain additional variance in models that also contained a Zika exposure variable (Fig 2). This finding was also robust to the lower than average birth size usually observed in the Northeast region (S2 Text, section 2.0) and the 15% reduction in birth rate that occurred during the Zika outbreak (S2 Text, section 2.1). ZIKV was also identified as the most likely cause when using aggregated data with an alternative (Poisson) model structure (S2 Text, section 2.2). We also found no evidence for the involvement of race of the mother in this association (S2 Text, section 2.3). Similar associations were found for MM and when using suspected Zika cases instead of PCR-confirmed Zika cases (S2 Text, section 2.4).

### Infection with ZIKV in the first 2 trimesters confers the greatest risk of MWSD

**Fig 3:**
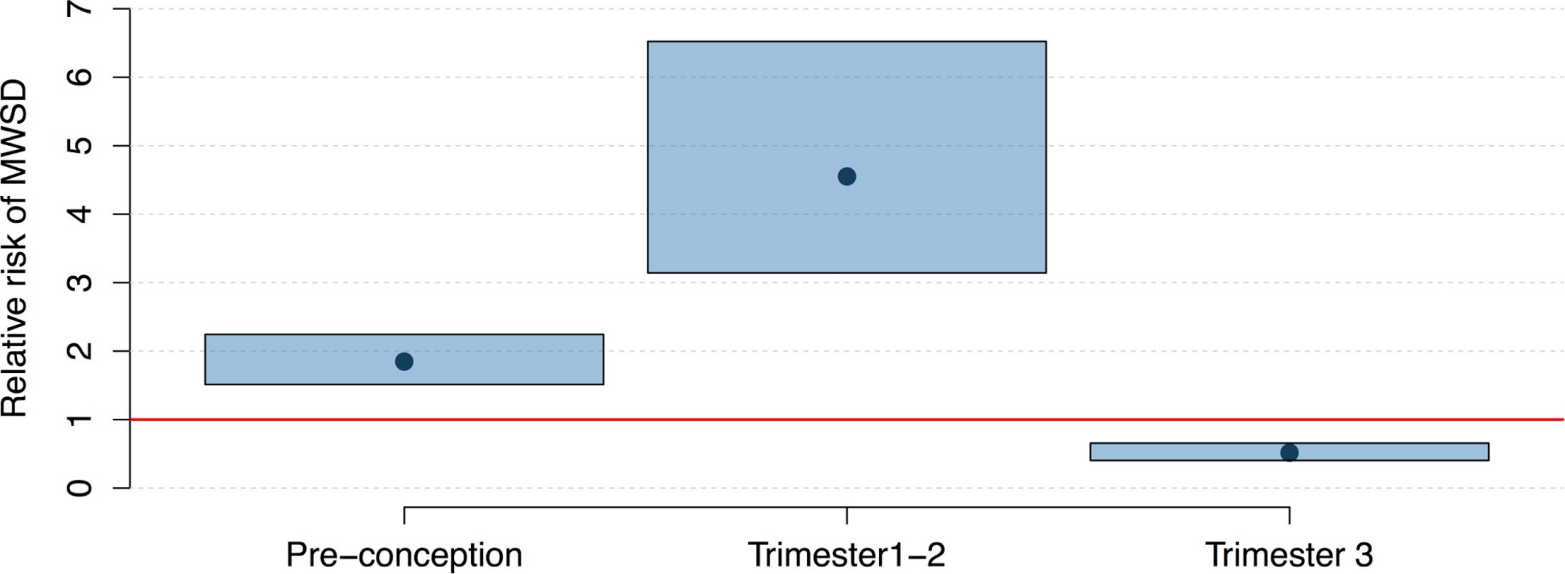
Relative risk of microcephaly with structural brain defects (MWSD) given Zika virus infection at different times in pregnancy. Black dots and blue bars represent the mean and 95% confidence intervals, respectively, of the estimated microcephaly relative risk for women exposed to median Zika virus incidence levels at different times in pregnancy. The red line shows a relative risk of 1 (i.e., no risk).

Disaggregation of the ZIKV–microcephaly association by timing of infection during pregnancy using the full exposure history dataset (Fig 1) showed that risk is concentrated in the first 2 trimesters of pregnancy (Fig 3). Due to collinearity between exposure in trimester 1 and trimester 2, we were unable to definitively state which conferred the higher risk. However, testing model combinations with either trimester 1 or trimester 2 omitted (S2 Text, section 2.5) gave some evidence that infection in the second trimester conferred a higher risk of MWSD.

We found that ZIKV infection preconception (up to 10 weeks) may also confer elevated risk, but at a lower level than infection in trimester 1 or 2. We found no association between third trimester infection and elevated risk of MWSD. The same findings were reached using MM (S2 Text, section 2.4), except relative risk was nonsignificant for preconception ZIKV infection.

### Estimating absolute and relative risk of MWSD given ZIKV infection in pregnancy

The time-specific exposure models provide the most precise estimate of the magnitude of the association between ZIKV and MWSD because it clearly defines exposure and includes a larger number of spatio-temporal covariates to appropriately capture the variation in MWSD due to background non-ZIKV causes, e.g., genetic, environmental, and TORCH(S) infections. Using the predictions from this model in the absence of any Zika suggested a mean national MWSD rate of 0.94 (95% CI 0.02–2.71) per 10,000 births, with the highest background rates in the Central-West and Northeast regions (S1 Text, section 1.7). When using the expanded definition of MM, this background rate increased to 1.80 (95% CI 1.77–1.82), which is likely to be a more comparable figure with ongoing microcephaly surveillance efforts outside Brazil [12]. Predicted baseline rates were also marginally higher in female babies (relative risk 1.25 [95% CI 1.05–1.48]), higher in the latter half of 2016 towards the end of the ZIKV outbreak, and higher in the northern state of Amapá, with the last 2 findings possibly due to increased surveillance efforts.

This would imply that 645 (95% CI 149–1,854) of the 2,791 MWSD cases were due to non-Zika background causes. Over the same time period 152 TORCH(S)-positive MWSD cases were reported; however, only a subset were tested (2,004 of 2,791 tested), and the test covered only a small number of potential causes of microcephaly, and may have been used at the wrong time in pregnancy.

Women whose first or second trimester of pregnancy occurred during a Zika outbreak had, on average, a 4.55 (95% CI 3.14–6.52) relative risk of MWSD compared to women pregnant before or after the outbreak (Fig 3); however, this relative risk varied considerably depending on the size of the ZIKV outbreak in the municipality of residence. To the best of our knowledge, only 1 study has measured true ZIKV attack rate in humans using population representative IgG seroprevalence surveys [30], finding an attack rate of 66% in the northeastern city of Salvador, Bahia. Adjusting our estimate for this municipality (Table 1) yields a relative risk of MWSD for an individual mother infected during pregnancy of 16.80 (95% CI 3.21–369.10).

**Table 1:** Estimated individual-level risk of MWSD.

Measure	Absolute MWSD risk per 10,000 births* or percentage probability of ZIKV infection (95% CI)	Relative risk of MWSD (95% CI)
For a pregnant woman living in Brazil (from current analysis**)	4.08 (2.63–7.24)	4.33 (0.97–332.12)
For a pregnant woman living in Salvador, BA (from current analysis**)	25.85 (22.85–29.25)	27.44 (8.44–1,340.95)
Percentage probability of ZIKV infection for a pregnant woman living in Salvador, BA (from [30])	63.3% (59.4%–66.8%)	—
For a pregnant woman infected*** with ZIKV in pregnancy (combining current analysis and [30])	40.84 (34.20–49.25)	16.80 (3.21–369.10)

*Absolute risk includes risk due to Zika and other baseline causes of MWSD.

**Current analysis estimates are from the time-specific exposure models (Fig 1) and compare model-predicted MWSD risk given the ZIKV experienced in each area compared to predicted risk with no ZIKV exposure.

***Infection includes both symptomatic and asymptomatic cases.

BA, Bahia; MWSD, microcephaly with structural brain defects; ZIKV, Zika virus.

**Fig 4:**
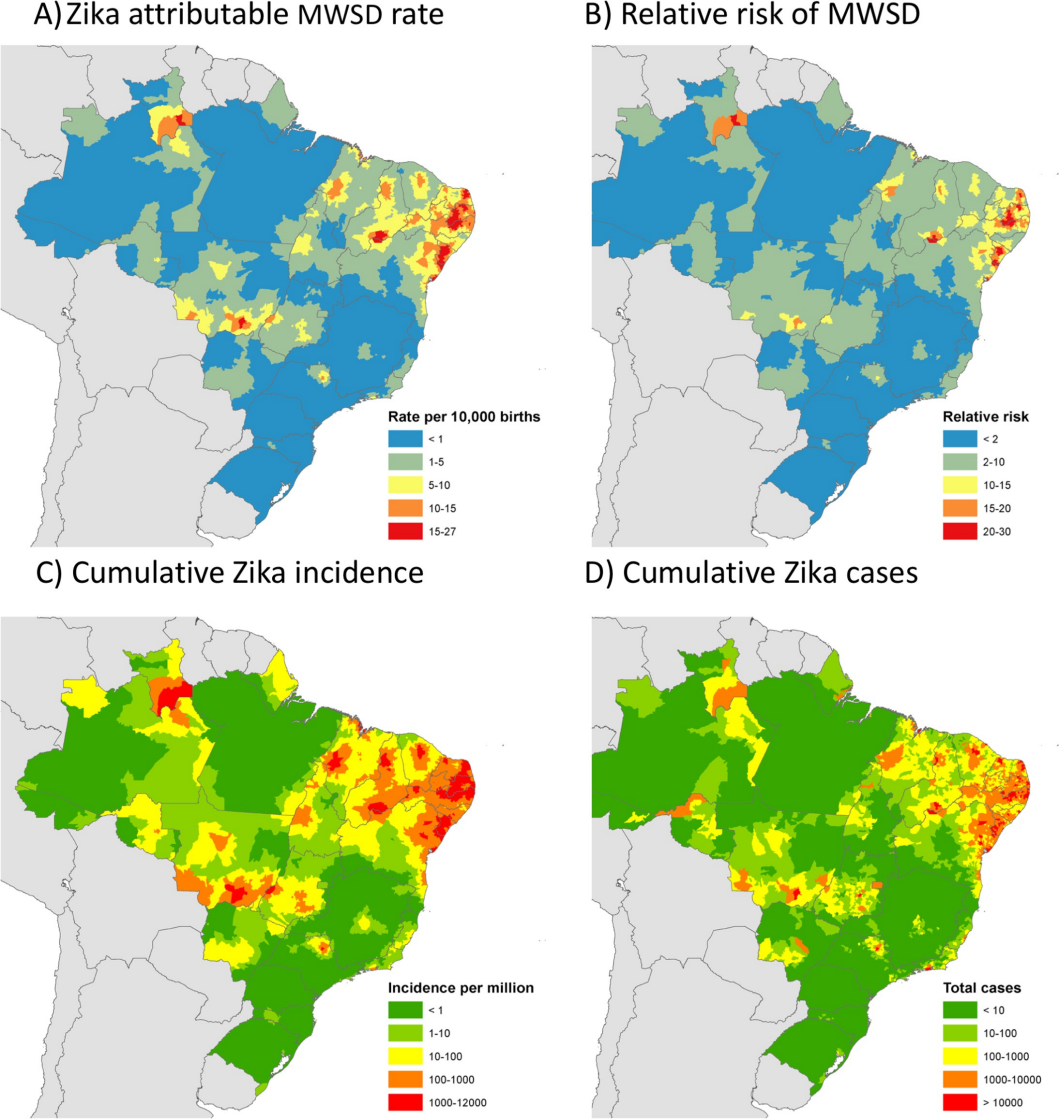
Concentration of the Zika virus (ZIKV) outbreak in Northeast Brazil. This figure shows (A) the ZIKV-attributable absolute rate of microcephaly with structural brain defects (MWSD) per 10,000 live births, (B) the relative risk of MWSD at birth given the ZIKV outbreak experienced in each municipality, (C) the model-predicted cumulative symptomatic Zika case incidence on a log 10 scale, and (D) the model-predicted total cumulative symptomatic Zika cases on a log 10 scale. All maps show municipality-level predictions averaged or summed over the time period January 2015–May 2017.

Mapping ZIKV-attributable MWSD rate and relative risk (Fig 4A and 4B) showed high heterogeneity in exposure and subsequent risk. Some women living in the Northeast region experienced a relative risk closer to 20–30, which corresponded to a rate of 15–27 MWSD cases per 10,000 births, whereas in most other areas rates were much lower.

### A consistent association suggests ZIKV disproportionately impacted Northeast Brazil

Regular and reliable Zika surveillance was not established until December 2015, by which time ZIKV had already been circulating in Brazil for 9–22 months [31–33], causing an unknown number of cases. Using the rate of reported MWSD cases and the ZIKV–microcephaly association quantified above, we estimated the number of cases that would have been identified had the surveillance system been in place since January 2015 and assuming Zika was the only cause of the excess microcephaly rates observed (i.e., no unmeasured confounding; S2 Text, section 2.5). This approach suggests 8.5 (95% CI 1.1–29.8) million symptomatic Zika cases have occurred in Brazil since the beginning of the outbreak (Table 2 and Fig 4D). Northeast Brazil has been the worst affected region, with 94% (95% CI 90%–97%) of total estimated cases. There was also considerable heterogeneity in incidence within the Northeast region, with the coastal states of Rio Grande do Norte, Paraíba, Pernambuco, Alagoas, and Sergipe worst affected. Some areas of the North region also experienced Zika incidence levels comparable to that of the Northeast region, particularly the state of Roraima (Fig 4C). Comparisons between estimated and reported cases (Table 2) suggest that only a small fraction (<1%) of the total Zika cases were reported in areas where the outbreak began, such as the Northeast region, whereas underreporting was much lower in areas that didn’t see significant transmission until later in the outbreak (e.g., Southeast region, 3% of cases reported).

**Table 2:** Estimated total symptomatic Zika cases over the course of the outbreak (January 2015–May 2017).

Region	Estimated cases	Percentage of burden	Incidence per 100,000 residents	Percentage of cases reported
Central-West	156,404 (11,288–812,226)	1.85 (1.04–2.72)	1,114.8 (80.5–5,789.5)	1.41 (0.27–19.52)
North	61,431 (4,967–326,294)	0.73 (0.46–1.09)	424.7 (34.4–2,256.1)	5.26 (0.99–65.01)
Northeast	7,940,825 (1,051,502–26,868,097)	93.73 (90.06–96.93)	15,171.8 (2,010.9–51,334.4)	0.02 (0.01–0.17)
South	7,187 (469–46,021)	0.08 (0.04–0.15)	26.3 (1.7–168.5)	2.34 (0.37–35.82)
Southeast	306,063 (16,607–1,779,332)	3.61 (1.53–5.96)	390.1 (21.2–2,267.6)	3.07 (0.53–56.57)
Total	8,471,910 (1,085,834–29,831,971)	100.00	4,539.9 (581.9–15,986.4)	0.20 (0.06–1.55)

Mean predictions with 95% confidence intervals in parentheses are shown.

### Zika also shows lower magnitude associations with some other congenital abnormalities detectable at birth

To test for any other potential congenital impacts of ZIKV, we repeated this test for association between ZIKV and different types of non-microcephaly congenital outcomes. This involved using the summary exposure history dataset with 5,360,851 births and 34,757 notified birth defects (Fig 1). These only included birth defects that were apparent and diagnosable at birth, were reported through routine birth reporting systems (SINASC), and were independent of the microcephaly reporting system (RESP).

**Fig 5:**
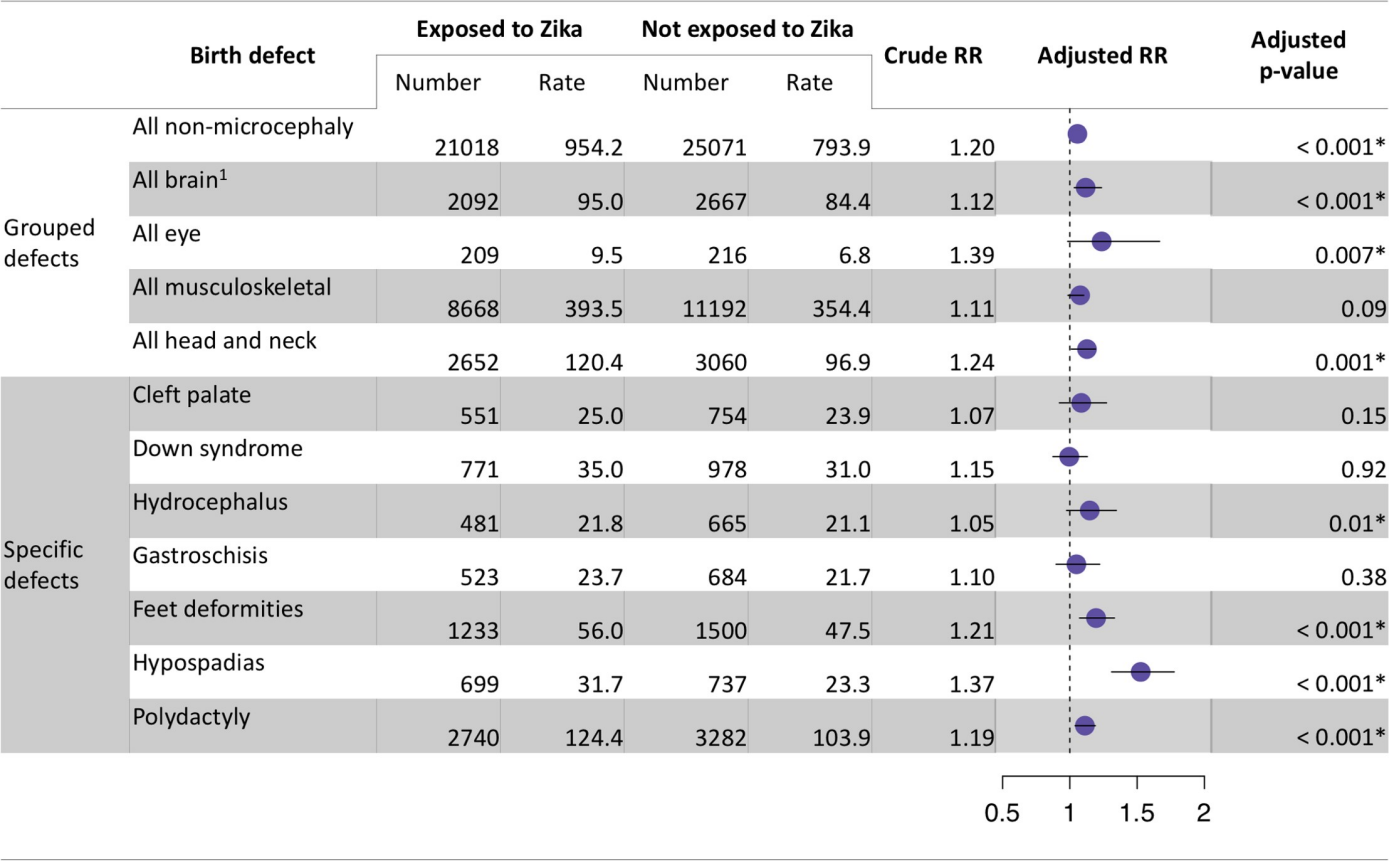
Relative risks of selected non-microcephaly birth defects with Zika virus. Exposure areas were defined as municipalities that experienced at least 1 case of Zika. Adjusted relative risk (RR) predictions show predictions for the median Zika incidence observed in Brazilian municipalities over the time period. Mean and 95% confidence intervals of relative risk are shown. The chi-squared test statistic was used with Bonferroni correction of *p*-values for 12 hypothesis tests. Significant results at the *p* = 0.05 level are denoted by an asterisk (*). Full ICD-10 categories for grouped defects are provided in S2 Text, section 2.6. ^1^All brain defects category excludes microcephaly.

After adjusting for non-ZIKV causes with the basic variables, we found a statistically significant association between Zika and polydactyly, hypospadias, feet deformities, hydrocephalus, and a larger group of eye, head and neck, and brain defects (chi-squared, all *p* < 0.01; Fig 5). In addition, musculoskeletal birth defects were also significant in the MM analysis (S2 Text, section 2.4). While statistically significant, the Zika-attributable rate and relative risk of these defects was considerably lower than that of microcephaly (1.1–1.5 versus 4.1; Fig 5 and Table 1), suggesting they were a rarer outcome of congenital ZIKV infection compared to microcephaly.

## Discussion

Here we present to our knowledge the first individual-level analysis of all births in Brazil since the beginning of the most recent ZIKV outbreak. We show that despite lower rates of microcephaly being reported outside Northeast Brazil, there was a consistent association between Zika and microcephaly that was not modified by arboviral effect modifiers or other environmental causes. Infection in the first 2 trimesters is likely to confer the greatest risk, with women living in average ZIKV outbreak areas subject to a 4.55 (95% CI 3.14–6.52) relative risk of MWSD and possible minor increased risk of other birth defects. This consistent association suggests that an estimated 8.5 (95% CI 1.1–29.8) million symptomatic Zika cases have occurred in Brazil since the beginning of the outbreak, with the majority (>90%) concentrated in the Northeast region.

A major limitation of this study is the use of an observational ecological measure of exposure, made necessary by the low rates of reliable ZIKV testing in pregnant women during the ZIKV outbreak. We have tried to limit the impact of this by using individual-level data to create a much higher spatio-temporal resolution match than is normally possible in ecological studies. One advantage of this ecological approach is its insensitivity to underreporting and the proportion of ZIKV infections that are symptomatic, if both remain consistent over space and time. This is important as a large proportion of Zika [34] and potentially microcephaly [35] cases are likely to have not been reported by routine surveillance. For our analysis, this may bias estimates of absolute risk, but not estimates of relative risk or strength of association. We were also able to translate our community-level estimates of relative risk to an individual level using data on ZIKV infection rates from the city of Salvador, estimating that a mother infected in pregnancy has a 16.80 (95% CI 3.21–369.10) relative risk of her child being born with MWSD. This individual-level relative risk overlaps with estimates from case–control—73 (95% CI 13–∞) [5]—and population-level observational studies—53.4 (95% CI 6.5–1061.2) [36] and 18–127 [9]. This analysis refines the evidence base that ZIKV infection in pregnancy is associated with an approximate 15- to 75-fold increase in microcephaly risk, highly depending on local baseline rate in the settings concerned. For the first time, to our knowledge, we also show that this association is consistent across a wide area including a range of settings.

Our results suggest that the ZIKV–microcephaly association was not modified by coinfection with dengue or chikungunya viruses, nor was it modified by prior vaccination for yellow fever. We were not able to test if dengue or chikungunya infection prior to pregnancy affected the ZIKV–MWSD relationship. Due to limitations of passive data including misdiagnosis and underlying immunity, prospective dengue cohort studies such as those established in Nicaragua [37] and Peru [38], where the timing of past exposure of individuals to dengue is well characterised, offer the best opportunity for investigating the effect of possible ZIKV–dengue virus cross-reaction on acute Zika disease severity. However, existing studies have not found strong correlations between Zika disease severity and the risk of congenital outcomes [39,40].

Throughout the Zika epidemic, there has been concern that microcephaly may have been the most apparent and most severe manifestation of a wide range of congenital abnormalities caused by ZIKV [41]. The evidence presented here contests this hypothesis, with a significant, high-magnitude association between ZIKV and microcephaly and a lack of any significant association or only low-magnitude associations between ZIKV and other common birth defects. While the specific links with eye defects, hypospadias, feet defects, and polydactyly merit further investigation, especially given the implications of some for fertility [42], these results suggest clinicians should focus principally on diagnosis of microcephaly in pregnant women with suspected ZIKV infection. However, these findings are based on a basic clinical assessment of newborns at the time of birth, and defects that only become apparent later in life will not have been recorded in these databases. Recent evidence suggests that up to 24% of microcephaly may only be diagnosable postnatally and that further developmental abnormalities may also occur [43]. Further longer term studies are needed in children born to ZIKV-infected (and non-ZIKV-infected) mothers to describe and measure the full lifetime burden of ZIKV, such as ongoing studies by the EU-funded ZIKAllliance, ZikaPLAN, and ZIKAction consortia.

A key weakness with retrospective data analyses, such as this, is the assumption of consistency in reporting of exposure and outcomes. One alternative explanation for our findings could be that the observed microcephaly epidemic may have, at least in part, been due to improved reporting of microcephaly given the high public profile of the ZIKV–microcephaly association at the time. Despite considerable differences in laboratory testing and MRI/CT scanning capacities in different parts of Brazil, we found no significant differences in testing rate or positivity rate between states in Brazil (S1 Text, section 1.1 and 1.2), probably due to the priority of Zika at the time and the high level of retrospective testing of samples. We also found no evidence of testing fatigue (where cases are no longer tested after the first confirmed case is reported; S1 Text, section 1.1.7). This means that even though not every suspected case of Zika or microcephaly was tested, the data are still a good representation of the profile of exposure and outcomes, supporting the validity of our assessment of association. We also (i) endeavoured to standardise all notifications to the latest, most specific case definition of microcephaly, (ii) included ‘time’ and ‘region’ variables to capture spatio-temporal variations in reporting, vector control efforts, and treatment-seeking behaviours, and (iii) performed sensitivity analyses using MM and suspected Zika cases, which gave similar results. Furthermore, no increases in the rate of other notified birth defects were observed over this time period (S2 Text, section 2.7). Despite this, there remains some significant spatial and temporal autocorrelation in the residuals of our best-fitting Zika model, in particular model overestimation of risk towards the end of the outbreak and in the state of Sao Paulo that could not be explained by known factors (S2 Text, section 2.8). These unexplained patterns could be related to Zika (e.g., complex changes in the way cases of the disease are reported) or due to some other unknown factor. Changes in surveillance practices would only bias our estimates of the magnitude of the association between Zika and microcephaly, not reduce our statistical confidence or our findings of Zika’s relative likelihood over other causes.

Because ZIKV surveillance was only established in December 2015, when the microcephaly outbreak had already begun, a major limitation of our study was our inability to assemble full exposure histories for over 3 million pregnancies, including for many of the MWSD cases from the first wave of the outbreak. This shaped our decision to assemble separate summary exposure history and full exposure history datasets. The full exposure history dataset may have been subject to selection bias as it disproportionately samples cases from the second wave of the epidemic and undersamples cases from the Northeast region. Aside from spatio-temporal differences, however, we found no age, sex, or socio-demographic differences between the population and selected records (S1 Text, section 1.2.1). The summary exposure dataset may suffer from misclassification of exposure due to the censored nature of the data; this was not formally accounted for in our model. Formally accounting for censoring in future modelling analyses using Bayesian hierarchical frameworks with multiple imputation for missing data may explain more of the spatio-temporal variation in MWSD and lead to more precise risk estimates for the ZIKV–MWSD association. We were also limited to the spatial scale of municipality for our analyses; variable municipal size across Brazil may introduce variation in spatial precision when mapping the rate of MWSD.

The observational nature of the analysis also means it may be subject to unmeasured confounding, especially given the multiple causes of microcephaly. In particular, we did not include exposure measures for TORCH(S) pathogens due to incomplete testing data. Our model-predicted baseline rate of 11% [4–32] of MWSD cases being due to non-ZIKV causes was comparable with the observed 8% of MWSD cases that were TORCH(S) positive among those who were tested. Due to computational constraints, we were not able to assess the stability of included baseline risk covariates through bootstrap sampling [44], which may limit our ability to accurately identify factors associated with the baseline risk of MWSD. Finally, we did not propagate record linkage uncertainty between the SINASC and RESP databases, which may have led to a small number of duplicates in our dataset and a very small bias towards the null, which could have been resolved with the use of multiple imputation.

We found that risk of MWSD was highest when ZIKV infection occurred in the first 2 trimesters of pregnancy. This is consistent with previous studies on ZIKV [6,36,39] and other acute infectious diseases where there is not only a hypothesized impact on the early fundamental stages of neurodevelopment, but also impacts that make subsequent development and maturation more likely to malfunction [45].

The finding that ZIKV infection prior to conception confers no elevated risk of microcephaly may be subject to survivor bias as we had no way to detect fetal demise with the databases used in this analysis. Fetal demise would also have the effect of underestimating relative risk of MWSD given ZIKV infection and underestimating the strength of association between these variables.

The strength of this analysis is its broad geographic scope and scale. We were able to analyse outcomes of between 3.6 and 5.9 million births in Brazil since 2015, allowing new levels of precision and generalisability in assessing rare congenital defects. With the now declining ZIKV epidemic limiting opportunities for new data collection, retrospective analyses of routinely collected data may provide the best possible approach to characterise the association between Zika and congenital abnormalities.

## Conclusions

This study adds further evidence that ZIKV infection in the early stages of pregnancy is associated with an elevated risk of microcephaly, with relative risk 16.80 (95% CI 3.21–369.10). Concentration of this risk in the first 2 trimesters of pregnancy suggests that women infected prior to conception or late in pregnancy are unlikely to be at excess risk. Here we found no evidence that the association between ZIKV and microcephaly was modified by arboviral exposure nor did we find strong evidence of alternative causes, and thus we hypothesize that the most likely reason why Northeast Brazil reported higher microcephaly rates was because it had more cases of Zika. We know that ZIKV circulated undetected, and thus uncontrolled, in the region for 9–22 months [31–33], and that year-round transmission suitability [46] in the Northeast region has historically led to higher dengue attack rates [47]. However, only seroprevalence surveys across the country can confirm this hypothesis and help us understand why the Northeast region was so adversely impacted. Such studies are vital for estimating the future risk of Zika and microcephaly outbreaks across the region.

## Supporting information

S1 Analysis plan The pre-approved statistical analysis plan. (DOCX)

S1 IRB Original submission to the London School of Hygiene &Tropical Medicine institutional review board. (PDF)

S1 STROBE STROBE checklist. (DOCX)

S1 Text Supplementary text describing data and data processing steps. (DOCX)

S2 Text Supplementary text describing supplementary analyses performed. (DOCX)

## Abbreviations

AIC: Akaike Information Criterion
BVDV: bovine viral diarrhoea virus
CNS: central nervous system
CT: computerised tomography
GAM: generalised additive model
MM: morphological microcephaly
MRI: magnetic resonance imaging
MWSD: microcephaly with structural brain defects
RESP: Registro de Eventos em Saúde Pública
SINASC: Sistema de Informações sobre Nascidos Vivos
TORCH(S): toxoplasmosis, other, rubella, cytomegalovirus, herpes, and syphilis
ZIKV: Zika virus
