# Supplementary material for: The association between Zika virus infection and microcephaly in Brazil 2015–2017: An observational analysis of over 4 million births
Source: PLoS Med. 2019 Mar 5;16(3):e1002755. doi: 10.1371/journal.pmed.1002755 (PMC6400331; doi:10.1371/journal.pmed.1002755)
Supplement: S1 Analysis plan — (DOCX) [file pmed.1002755.s001.docx]

**APPENDIX 1: TEMPLATE: STATISTICAL ANALYSIS PLAN
(Use headings as appropriate)**

**Statistical Analysis Plan**

**<Name of Trial>**

**FINAL VERSION APPROVED BY:**

| **Name** | **Signature** | **Date** |
| --- | --- | --- |
|  |  |  |
|  |  |  |

**CURRENT VERSION APPROVED BY (IF CHANGED FROM FINAL):**

| **Name** | **Signature** | **Date** |
| --- | --- | --- |
| Oversight body |  |  |
| Trial Statistician | **Oliver Brady** | **22/11/2017** |

| **REVISION OF FINAL VERSION**  **CHRONOLOGY:** | | | |
| --- | --- | --- | --- |
| **VERSION NUMBER** | **EFFECTIVE DATE** | **REASON FOR CHANGE** | **CHANGE APPROVED BY (NAME)** |
|  |  |  |  |
|  |  |  |  |
|  |  |  |  |

**INDEX**

[1. STUDY SYNOPSIS](javascript:__doPostBack()

This study aims to test for and characterise the association between Zika and microcephaly using a retrospective analysis of live birth data from over 4 million women in Brazil

[2. STUDY OBJECTIVES](javascript:__doPostBack()

[2.1 Primary Objective](javascript:__doPostBack()

To test for an association between Zika incidence in area of residence at the time of pregnancy and increased odds of microcephaly at birth

[2.2 Secondary Objectives](javascript:__doPostBack()

To test for potential cofactors / effect modifiers of this association

To test for an association between Zika incidence in area of residence at the time of pregnancy and increased odds of other non-microcephaly birth defects

[2.3 Assessment of Objectives](javascript:__doPostBack()

Penalized Logistic regression with selection of potential confounders (socioeconomic status, time, region) selected by AIC. Different primary exposures (Zika, Dengue, Chikungunya, water toxins, bovine exposure and interactions between Zika and dengue and chikungunya) will be evaluated by relative AIC when compared to a confounder only model.

Tests for other birth defects will also include a backwards confounder selection by AIC step with the final Zika exposure term evaluated using p-values at the 99% level given the size of the dataset.

[2.4 Change the Primary Objective During the Conduct of the Study](javascript:__doPostBack()

No

[3. STUDY DESIGN](javascript:__doPostBack()

[3.1 General Design and Plan](javascript:__doPostBack()

Retrospective analysis of individual level birth outcomes linked to ecological measures of exposure (matched by municipality of residence and timing of pregnancy)

[3.2 Sample Size](javascript:__doPostBack()

NA – retrospective analysis to test for association
[3.3 Randomization and Blinding](javascript:__doPostBack()

NA
[3.4 Study Assessments](javascript:__doPostBack()

NA

[4. STUDY POPULATIONS](javascript:__doPostBack()

[4.1 Subject Disposition](javascript:__doPostBack()

NA
[4.2 Definition of Populations for Analysis](javascript:__doPostBack()

Every women registered as having a live birth over the time period an 2015 to May 2017
[4.3 Efficacy Evaluable](javascript:__doPostBack()

NA
[4.4 Intent-to-treat (ITT)](javascript:__doPostBack()

NA
[4.5 Per Protocol (PP)](javascript:__doPostBack()

NA
[4.6 Safety Population](javascript:__doPostBack()

NA
[4.7 Major Protocol Deviations](javascript:__doPostBack()

NA
[4.8 Definition of Sub-Group Population in Different Analyses](javascript:__doPostBack()

Analysis will only be conducted on women for which all information on exposure to potential confounders and all candidate exposures can be assembled

[5. STATISTICAL ANALYSIS](javascript:__doPostBack()

[5.1 General](javascript:__doPostBack()

Penalised logistic regression at the individual birth level
[5.2 Pooling of Sites](javascript:__doPostBack()

NA
[5.3 Interim Analyses](javascript:__doPostBack()

NA
[5.4 Time-Points For Analysis](javascript:__doPostBack()

Jan 2015- May 2017

[5.5 Methods for Handling Missing Data](javascript:__doPostBack()

Omission

[5.6 Statistical Analytical Issues](javascript:__doPostBack()

NA

[6. EVALUATION OF DEMOGRAPHICS AND BASELINE CHARACTERISTICS](javascript:__doPostBack()

[6.1 Demographics and Baseline Characteristics](javascript:__doPostBack()

Data is from a National registry, so population-representative of Brazilian demographics
[6.2 Medical History and Prior Medical Therapy](javascript:__doPostBack()

NA
[6.3 Prior Therapies and Medications](javascript:__doPostBack()

NA

[7. EVALUATION OF TREATMENT COMPLIANCE AND EXPOSURE](javascript:__doPostBack()

[7.1 Compliance to Study Drug and Treatment](javascript:__doPostBack()

NA
[7.2 Exposure to Study Drug](javascript:__doPostBack()

NA

[8. EVALUATION OF PHARMACOKINETICS](javascript:__doPostBack()

[8.1 Evaluation of Pharmacokinetics](javascript:__doPostBack()

NA
[8.2 Pharmacokinetic Parameters](javascript:__doPostBack()

NA
[8.3 Bioequivalent](javascript:__doPostBack()

NA

[9. EVALUATION OF EFFICACY PARAMETERS](javascript:__doPostBack()

[9.1 Analysis of Primary, Secondary, and Other Efficacy Endpoints](javascript:__doPostBack()

NA
[9.2 Method for Analysis of Efficacy Endpoints](javascript:__doPostBack()

NA

[10. EVALUATION OF SAFETY PARAMETERS](javascript:__doPostBack()

[10.1 Adverse Events](javascript:__doPostBack()
NA

11 REFERENCE

12 PROTOCOL VIOLATIONS
