# Supplementary material for: The association between Zika virus infection and microcephaly in Brazil 2015–2017: An observational analysis of over 4 million births
Source: PLoS Med. 2019 Mar 5;16(3):e1002755. doi: 10.1371/journal.pmed.1002755 (PMC6400331; doi:10.1371/journal.pmed.1002755)
Supplement: S1 IRB — (PDF) [file pmed.1002755.s002.pdf]

LSHTM Ethics Application & CARE Form

Project Information

1. Full project title

The association between Zika and microcephaly in pregnant women in Brazil

2. Is this Project in fulfillment of a degree?

- ○ Yes
- ◉ No

2a. Is this project research?

- ◉ Yes
- ○ No

2f(staff). Is this an original submission, or are you responding to a request for clarification from the LSHTM ethics committee?

- ◉ Original submission
- ○ Responding to request for clarification

Applicant Details

3a. Details for LSHTM lead investigator

Title	First Name	Surname
Dr	Oliver	Brady

Address	Keppel Street
City	London
Postcode	WC1E 7HT
Telephone	00447814561078
Email	oliver.brady@lshtm.ac.uk

3b. Job title of LSHTM Lead Investigator

Assistant Professor

3c. Faculty of LSHTM Lead Investigator

Epidemiology and Population Health (EPH)

3d. Department of LSHTM Lead Investigator

Department of Infectious Disease I

3e. Are you the Chief Investigator for the research project?

- ○ Yes
- ◉ No

3e (i). Name of Chief Investigator (CI)

Prof Simon Hay

3e (ii). Institution of Chief Investigator (CI)

University of Washington, WA, USA

3f. Other personnel involved

Dr. Fatima Marinho, director of information and health analysis at Brazilian ministry of health

Project Type

Note: Completing the filter will enable and disable sections of the form so you may not see all questions.

4. Does the research involve primary data collection, secondary analysis or a mix of both?

- ○ Primary
- ◉ Secondary
- ○ Mixed

4a(iii). Select type of project:

Project using data from secondary sources

Samples

6a. Does this research project involve the collection/use of human tissue samples e.g urine, stool, blood etc? (Please select yes even if the samples are not considered relevant material under the Human Tissue Act)

- ( ) Yes
- (•) No

6b. Will this project use living animals (either laboratory, livestock or wild animals) AND/OR biological material that has been obtained from animals in the experiments planned?

- ( ) Yes
- (•) No

Fast-Track

7. Does this project use anonymised and unlinkable secondary datasets only?

- (•) Yes
- ( ) No

7a. Will this project be conducted within the NHS?

- ( ) Yes
- (•) No

7b. Is this application for fast-track? Note: MSc applications are not currently available for fast-track

- (•) Yes
- ( ) No

7c. Select reason for fast-track

Using anonymised and unlinkable secondary datasets only

Vulnerable Groups

8c. Does this research project involve vulnerable groups? Vulnerable groups include: children, individuals with mental disability or learning difficulties, pregnant women, prisoners etc (see information icon for full description).

- [x] Yes
- [ ] No

8c(i) Please tick the group(s) below

- [ ] Children
- [ ] Individuals with mental disability or learning difficulties
- [x] Pregnant women
- [ ] Prisonsers
- [ ] Unconscious or severely ill
- [x] Foetuses and neonates
- [ ] Staff or students of LSHTM
- [ ] Other

8d. Does this research involve access to and/or storage of security sensitive research material? (please see information icon for what is considered security sensitive material)

- [ ] Yes
- [x] No

Geography

9. List the countries where the research project is to be conducted (For example: if you are conducting a secondary data analysis for your project and you will be based in the UK, select UK regardless of where the original data has come from):

United Kingdom

9. List the countries where the research project is to be conducted (For example: if you are conducting a secondary data analysis for your project and you will be based in the UK, select UK regardless of where the original data has come from):

United States of America

Please be aware that all primary health research conducted in the UK requires a sponsor. Please contact the RGIO at RGIO@lshtm.ac.uk for more information on sponsorship.

Outline

Note: Please do not copy and paste directly from the protocol. Applications where large portions of text have been copied and pasted directly from the protocol, and therefore do not properly answer the question, will be invalidated

10b. Give an outline of the proposed project, including background to the proposal. Include information from any systematic reviews that have been conducted. Sufficient detail must be given to allow the Committee to make an informed decision without reference to other documents.

In 2015 Brazil reported an increase in the congenital abnormality microcephaly in northeast Brazil that coincided with the first introduction of Zika into Brazil. At the time the association was difficult to prove at the population level as routine Zika surveillance was not establishing in Brazil until November 2015. Now sufficient time has passed since the establishment of Zika surveillance and the completion of around 4 million pregnancies that the association between Zika and microcephaly can be quantified at the population level. Because of the rarity of microcephaly, prospective maternal cohort studies have struggled to reach adequate sample sizes to conclusively test this association, particularly with regards to potential cofactors / effect modifiers. As a result retrospective observational case control studies using routinely collected data can give new insights into this association.

The majority of the data used in this analysis is fully publicly available through Brazilian ministry of health websites. However a few key details are needed to analyse this association at the individual level. These include i) congenital abnormalities detected at birth (ICD-10 coded - the response variable), ii) date of birth, gestational age at birth and municipality (2nd Administrative level, county) of residence of the mother (to match period of potential exposure) and iii) mother age, race, sex of the baby (to adult for potential confounders). All of this individual level information is routinely collected at birthing entered into the SINASC database of all births in Brazil .We will be analysing fully anonymised individual records with only the above information.

Logistic regression at the level of individual pregnancy will be used to test the association between Zika outbreaks in area of residence during pregnancy and microcephaly risk at birth. Only summary statistics will be presented in the final publication and no individual level data will be published.

10b(i). Upload the study protocol, including data collection forms, questionnaires and topic guides. Please upload each document separately, ensuring that the date and version number of each document is correct.

Type	Document Name	File Name	Version Date	Version	Size
Protocol / Proposal	Ethics_SAP	Ethics_SAP.docx	22/11/2017	1	30.3 KB

11. State the intended value of the project, detailing why the topic is of interest or relevance. If this project or a similar one has been done before what is the value of repeating it? Give details of overviews and/or information on the Cochrane database. This area is of increasing importance – please ensure you give a full response.

Quantifying the association between Zika infection in pregnancy and microcephaly remains a key unknown, particularly in Brazil where rate of microcephaly have been significantly higher in the northeast of the country than in the southern states. This has led some to suggest the involvement of cofactors or alternative causes. We will be able to test these hypotheses with this data and analysis plan. We will also be able to provide further evidence on what times ZIKV infection in pregnancy leads to elevated risk of microcephaly which can be used to give better advice to women who live in affected areas. Finally we will test the association between Zika and other potential congenital abnormalities to better characterise the burden of disease.
This is a common aim of a number of EU Zika consortia (ZikAlliance, ZikaPlan and ZikAction) but the decline of the Zika epidemic in 2017 has meant some sites are struggling to recruit women whoa re infected with Zika in pregnancy. As a result retrospective analysis of routinely collected data can offer a novel contribution to characterising key metrics of the association between Zika and microcephaly.

13. Overall aim of project

To characterise the association between Zika virus infection in pregnancy and the risk of congenital abnormalities at birth

14. Specific objectives of project

1. To test the likelihood of Zika and other candidate exposures in isolation and as cofactors in explaining the observed increased incidence rate of microcephaly in Brazil
2. To identify in which gestational weeks of pregnancy Zika infection confers the greatest risk of microcephaly
3. To test for an association between maternal Zika infection and other non-microcephaly congenital abnormalities

Methods

Note: Please do not copy and paste directly from the protocol. Applications where large portions of text have been copied and pasted directly from the protocol, and therefore do not properly answer the question, will be invalidated

15a. Specify the procedures/methodology to be conducted during the project. Please include outcome measures and plans for data management and analysis. For literature reviews, include details on search strategy, search terms, inclusion and exclusion criteria.

Logistic regression will be used to test the association between a number of ecological exposure variables and microcephaly at birth. Potential confounders including individual level mother age, race and sex of the baby will also be included and selectively removed by backwards section using AIC.

For objective 1(cause and cofactors of microcephaly), different candidate models will be evaluated using likelihood ratio tests and change in relative risk

For objective 2 (timing of Zika infection and risk of microcephaly) thin plate spline based statistical models will be used to fit a relationship between timing of zika infection (in gestational weeks) and relative risk of microcephaly.

For objective 3 (Zika association with other birth defects), the significance of the coefficient of the Zika covariate in the confounder adjusted model will be tested using the chi squared test statistic

All data will be stored only on LSHTM hard drives, will be encrypted and will have a hard backup physically located within LSHTM in London.

16. Proposed start date of the project

30/11/2017

17. Proposed end date of the project

30/12/2017

Experience

22a. State the personal experience of the applicant and of senior collaborators in the research project in the field concerned, and their contribution to this project. Indicate any previous work done related to the project topic including student and/or professional work, or publications

I have significant experience of secondary data analysis at the population level including the use of Brazilian infectious disease data (see Brady et al. 2015 Epidemics 11, 92-102). I will be responsible for conducting all statistical analyses.

Prof. Hay (http://www.healthdata.org/about/simon-iain-hay) has significant experience working wth both routinely collected ministry of health databases and survey data (see http://www.map.ox.ac.uk). He will supervise the project and help interpret the results

Dr. Fatima Marinho is director of surveillance at the Brazilian Ministry of Health whose primary responsibility is secondary data analysis of epidemiological data. She will provide insights into how the data were collected and help interpret the results.

22b. Upload the CVs for all main investigators working on the project. For MSc students, please upload your CV only.

Type	Document Name	File Name	Version Date	Version	Size
Investigator CV	LSHTM_CV_OJB_AUG17	LSHTM_CV_OJB_AUG17.docx	01/11/2017	1	78.0 KB

Informed Consent - Secondary Data

24. Is consent in place for secondary use of the data?

- (x) Yes
- ( ) No

24c(i). Please give details of the participant consent that was obtained when the original project(s) took place. Please upload copies of the original consent form(s). If there are no original consent forms (e.g. for audit or DHS data) please explain this.

Data is routinely collected at birth and entered into a database of all births in Brazil (SINASC). This project only deals with processed secondary anonymised data from this database and has no access to original consent forms.

Confidentiality & Data

28. State how your data will be stored and what will be done with it at the end of the project.

Data will be stored only on LSHTM hard drives, will be encrypted and will have a hard backup that will stay physically within the school.

Funding

33. Do you have external funding for this project?

- (x) Yes
- ( ) No

33a. If yes, please provide the name of the funder

European Union Horizon 2020, Wellcome Trust

33a(i). If yes, include details of the funding available for this project.

ZikAlliance consortium, Henry Wellcome Fellowship

33a(ii). Are you in receipt of any funding from the United States? Or will you be collaborating with (or with individuals from) a US Institution/organisation?

- (•) Yes
- ( ) No

34. Has the project been sent out for peer/independent scientific review?

- (•) Yes
- ( ) No

34b. If yes, who has provided peer/independent scientific review of the project?

> Faculty at IHME, University of Washington (the lead institution).

36. Does the Chief Investigator or any other investigator/collaborator have any direct personal involvement (e.g. financial, share holding, personal relationship etc.) in the organisations sponsoring or funding the research that may give rise to a possible conflict of interest?

- ( ) Yes
- (•) No

37. Will individual researchers receive any personal payment over and above normal salary, or any other benefits or incentives, for taking part in this research?

- ( ) Yes
- (•) No

Local Approval

49a. For projects using previously-collected human data, give details of all approvals under which the original project(s) took place. Please quote names of Ethics Committees and approval reference numbers (required even if previous approval was from LSHTM); if possible give web link to original project application. If there are no original approvals (e.g. for audit or DHS data) please explain this.

> This project uses data routinely collected by the Brazilian Ministry of Health in the form of basic birth details about the mother and pregnancy recorded at birth. This data is permitted to be used for secondary anonymised epidemiological analyses, such as the project proposed here.

Type	Document Name	File Name	Version Date	Version	Size
Local Approval	Data_permissions	Data_permissions.pdf	16/11/2017	1	890.2 KB

49c. Will your analyses be for purposes entirely covered by the original ethics application where the data was collected, as detailed above?

- ( ) Yes, this falls within the aims and scope of the original project
- (•) No, the analyses and aims differ from the original project

49d(ii). If no, please detail how you will amend the original ethics application to include the current analysis.

See local approval form attached

Signature Instructions

The form should be completed and finalised prior to signing or requesting signatures. Students should ensure that the Supervisor signs prior to the Course Director/Project Module Organiser. For external supervisors, please ensure that they have registered for an account prior to requesting the signature.

Signature - Applicant

LSHTM Lead Investigator

I declare that:

- **The information in this form is accurate to the best of my knowledge and I take full responsibility for it**
- I undertake to abide by the ethical principles underlying the Declaration of Helsinki (1964, as amended) and good practice guidelines on the proper conduct of research.
- I Have read and understood, and agree to abide by the LSHTM Good Research Practice policy
- I undertake to abide by the Data Protection Act 1998 and any applicable local laws.
- I undertake to abide by all local rules for non-UK research.
- I will report any unexpected serious adverse reactions or other serious unforeseen events which occur to the LSHTM Ethics Committee promptly.
- I will provide an annual progress report until the end of the research.
- I will provide notification of the end or early termination of the research project.
- I will inform the LSHTM Ethics Committee if there are any changes to the research protocol or personnel which affect the ethical aspects of the project.
- I will assist LSHTM Ethics Committee in any continuing review of the project deemed necessary by the Committee or Faculty members.
- I undertake to adhere to the project protocol, the terms of the full application as approved and any conditions set out by review bodies in giving approval and will not start the project until all required approvals are in place.
- I confirm that there are no conflicts of interest that preclude my participation in the study

Signed: This form was signed by Dr. Oliver Brady (Oliver.brady@lshtm.ac.uk) on 22/11/2017 11:11 PM

Signature - Other

Note:

The form will automatically submit upon receipt of all required signatures.

After submission, you will receive a confirmation email with further details.

If you have not received a confirmation email within 5 working days please email ethics@lshtm.ac.uk (staff) or MScethics@lshtm.ac.uk (students) to check the status of your submission.
