# Supplementary material for: The association between Zika virus infection and microcephaly in Brazil 2015–2017: An observational analysis of over 4 million births
Source: PLoS Med. 2019 Mar 5;16(3):e1002755. doi: 10.1371/journal.pmed.1002755 (PMC6400331; doi:10.1371/journal.pmed.1002755)
Supplement: S1 STROBE — (DOCX) [file pmed.1002755.s003.docx]

# S1 STROBE

STROBE Statement—Checklist of items that should be included in reports of ***case-control studies***

|  | Item No | Recommendation |
| --- | --- | --- |
| **Title and abstract** | 1 | *(*a) Indicate the study’s design with a commonly used term in the title or the abstract  *DONE- described in the title as “an observational analysis”* |
|  |  | (*b*) Provide in the abstract an informative and balanced summary of what was done and what was found  *DONE- we,*  *i) describe the main analysis performed: “The association between ZIKV and microcephaly was then statistically tested against models with alternative causes or with effect modifiers”*  *ii) describe what was found: “We found no evidence for alternative non-ZIKV causes of the 2015-2017 MWSD outbreak, nor that concurrent or previous exposure to arbovirus infection or vaccination modified risk”*  *iii) include a limitation identifying what we were not able to find:* “Our analysis was limited by missing data prior to establishment of nationwide ZIKV surveillance and its findings may be affected by unmeasured confounding causes of microcephaly no available in routinely collected surveillance data” |
| Introduction | | |
| Background/rationale | 2 | Explain the scientific background and rationale for the investigation being reported  *DONE- in paragraph 1 we describe the public health context of the microcephaly outbreak, paragraph 2, describes existing evidence of its association with ZIKV, paragraph 3 gives background knowledge on microcephaly and identifies key challenges in assessing its association with ZIKV. Paragraph 5 identifies alternative hypotheses and paragraph 6 builds the rationale for our overall aims and why we chose to answer them using the approach we did.* |
| Objectives | 3 | State specific objectives, including any prespecified hypotheses  *DONE – In the last paragraph of the introduction, we state: “We then use this large dataset to statistically compare different hypotheses for the observed pattern of microcephaly with the aim of characterising the magnitude and significance of the ZIKV-microcephaly association”. Hypotheses are outlined in paragraph 5 of the introduction.* |
| Methods | | |
| Study design | 4 | Present key elements of study design early in the paper  *DONE – In the last paragraph of the introduction, we state the key features of the study ( that it is individual , level observational and uses ecological measures of exposure)* |
| Setting | 5 | Describe the setting, locations, and relevant dates, including periods of recruitment, exposure, follow-up, and data collection  *DONE – In first, second and third paragraph of the Methods section the setting (Brazil) is described along with data time periods (including changes in definitions of microcephaly and ZIKV diagnosis). SA an analysis of secondary data no recruitment, follow up or collection dates were relevant.* |
| Participants | 6 | *(*a) Give the eligibility criteria, and the sources and methods of case ascertainment and control selection. Give the rationale for the choice of cases and controls  *DONE – In the second paragraph of the methods the eligibility and case ascertainment for RESP cases are described. In the third paragraph it is clearly stated that cases in the RESP database form cases in our analysis, while non-microcephaly births without a RESP record form controls.* |
|  |  | (*b*) For matched studies, give matching criteria and the number of controls per case  *NA Not a matched study* |
| Variables | 7 | Clearly define all outcomes, exposures, predictors, potential confounders, and effect modifiers. Give diagnostic criteria, if applicable  *DONE –Third paragraph of the methods defines outcomes. Exposures are defined in paragraphs 4, 5 and 6. Diagnostic criteria for ZIKV exposure are defined in paragraph 4.* |
| Data sources/ measurement | 8* | For each variable of interest, give sources of data and details of methods of assessment (measurement). Describe comparability of assessment methods if there is more than one group  *DONE – Data sources are given in the first paragraph of the methods including details of measurement. Further details are given in S1 Text, section 1.1 and 1.2. The analysis is repeated for “confirmed” and “suspected” cases with the alternative results given in S2 Text, section 2.4.* |
| Bias | 9 | Describe any efforts to address potential sources of bias  *DONE –In the third and fourth paragraph of the methods data process procedures are described to remove bias in microcephaly and ZIKV case data, such as temporal standardisation* |
| Study size | 10 | Explain how the study size was arrived at  *No formal study sample size calculations were completed due to the scale of the dataset > 4 million records in the majority of analyses.* |
| Quantitative variables | 11 | Explain how quantitative variables were handled in the analyses. If applicable, describe which groupings were chosen and why  *DONE – In paragraph six of the methods quantitative variable processing is described (zero inflation and log transformation).* |
| Statistical methods | 12 | *(*a) Describe all statistical methods, including those used to control for confounding  *DONE –All statistical methods are described in paragraphs 9-18 of the methods including stratification by all of the main sub-analyses* |
|  |  | (*b*) Describe any methods used to examine subgroups and interactions  *DONE –Sub analyses on the timing of risk are presented in the methods section titled “Assessing timing of risk during pregnancy”* |
|  |  | (*c*) Explain how missing data were addressed  *DONE – Missing data and data selection are described in paragraph 7 and 8 of the methods* |
|  |  | (*d*) If applicable, explain how matching of cases and controls was addressed  *NA* |
|  |  | (*e*) Describe any sensitivity analyses  *DONE – The analysis is repeated using suspected rather than confirmed microcephaly and ZIKV cases with the full results presented in S2 Text, section 2.4 which is discussed in fourth paragraph of the results section.* |
| Results | | |
| Participants | 13* | (a) Report numbers of individuals at each stage of study—eg numbers potentially eligible, examined for eligibility, confirmed eligible, included in the study, completing follow-up, and analysed  *DONE- reported in figure 1 and numbers analysed are repeated at the beginning of each section of the results* |
|  |  | (b) Give reasons for non-participation at each stage  *DONE- the inclusion criteria for each data subset are discussed in the first paragraph of each analysis step and some discussion of potential selection biases is included* |
|  |  | (c) Consider use of a flow diagram  *DONE- figure 1* |
| Descriptive data | 14* | (a) Give characteristics of study participants (eg demographic, clinical, social) and information on exposures and potential confounders  *DONE- Study uses national databases that are population representative of births in Brazil. We describe any differences in birth and mother characteristics (exposures and potential confounders) between this database and the final data used in the analyses in Supplementary information section 1.2.1.* |
|  |  | (b) Indicate number of participants with missing data for each variable of interest  *DONE – given in figure 1 and final numbers for each analysis given in the main results text section.* |
| Outcome data | 15* | Report numbers in each exposure category, or summary measures of exposure  *DONE- for all birth defects analysis this is combined with the results figure (figure 5), for the hypothesis testing analysis. This is now included in the introductory section of the results section.* |
| Main results | 16 | *(*a) Give unadjusted estimates and, if applicable, confounder-adjusted estimates and their precision (eg, 95% confidence interval). Make clear which confounders were adjusted for and why they were included  *DONE- given in figures 2 and 5* |
|  |  | (*b*) Report category boundaries when continuous variables were categorized  *NA- no continuous variables categorized* |
|  |  | (*c*) If relevant, consider translating estimates of relative risk into absolute risk for a meaningful time period  *DONE- see table 1* |

| Other analyses | 17 | Report other analyses done—eg analyses of subgroups and interactions, and sensitivity analyses  *DONE- we report the sensitivity of various findings to multiple sensitivity analyses documented in the supplementary information (sections 1.8, 1.9, 2.0, 2.1, 2.2, 2.4).* |
| --- | --- | --- |
| Discussion | | |
| Key results | 18 | Summarise key results with reference to study objectives  *DONE- summarised in first paragraph* |
| Limitations | 19 | Discuss limitations of the study, taking into account sources of potential bias or imprecision. Discuss both direction and magnitude of any potential bias  *DONE- discussed multiple times throughout the discussion* |
| Interpretation | 20 | Give a cautious overall interpretation of results considering objectives, limitations, multiplicity of analyses, results from similar studies, and other relevant evidence  *DONE- limitations outlined for every analysis, other studies referenced on ZIKV-microcephaly relative risk estimates and follow-up studies (Seroprevalence) suggested to test hypotheses raised in this paper* |
| Generalisability | 21 | Discuss the generalisability (external validity) of the study results  *DONE- with reference to the variability in background MWSD rate and the variability of ZIKV relative risk within Brazil.* |
| Other information | | |
| Funding | 22 | Give the source of funding and the role of the funders for the present study and, if applicable, for the original study on which the present article is based  *DONE- given in manuscript submission online system* |

*Give information separately for cases and controls.

**Note:** An Explanation and Elaboration article discusses each checklist item and gives methodological background and published examples of transparent reporting. The STROBE checklist is best used in conjunction with this article (freely available on the Web sites of PLoS Medicine at http://www.plosmedicine.org/, Annals of Internal Medicine at http://www.annals.org/, and Epidemiology at http://www.epidem.com/). Information on the STROBE Initiative is available at http://www.strobe-statement.org.
