# Supplementary material for: The association between Zika virus infection and microcephaly in Brazil 2015–2017: An observational analysis of over 4 million births
Source: PLoS Med. 2019 Mar 5;16(3):e1002755. doi: 10.1371/journal.pmed.1002755 (PMC6400331; doi:10.1371/journal.pmed.1002755)
Supplement: S1 Text — (DOCX) [file pmed.1002755.s004.docx]

S1 Text to:

“The association between Zika and microcephaly: an observational analysis of over four million births in Brazil 2015-2017”

Table of Contents

[SI1.1: Arbovirus data description 2](#_Toc535917146)

[SI1.1.1 Variation over time of suspected and confirmed cases 3](#_Toc535917147)

[SI1.1.2. Variation over space of suspected cases 4](#_Toc535917148)

[SI1.1.3. Distribution of total cases at the municipality level 4](#_Toc535917149)

[SI1.1.4. Distribution of outbreak duration at the municipality level 6](#_Toc535917150)

[SI1.1.5. Testing and confirmation rates (total) 6](#_Toc535917151)

[SI1.1.6. PCR confirmation rates by state 7](#_Toc535917152)

[SI1.1.7. Testing fatigue analysis 8](#_Toc535917153)

[SI1.2: Microcephaly data description 10](#_Toc535917154)

[SI1.2.1 Assessing selection bias by comparing the distribution of key characteristics between data sub-selections 11](#_Toc535917155)

[SI1.3: Drinking water quality index construction 13](#_Toc535917156)

[SI1.4 Missing data 14](#_Toc535917157)

[SI1.5: Socio-demographic Index 15](#_Toc535917158)

[SI1.6: Record linkage algorithm to match records in RESP to records in SINASC 15](#_Toc535917159)

[SI1.7: Estimating the baseline rate of microcephaly 16](#_Toc535917160)

[SI1.8: Fit of GAM smoother to microcephaly data used in microcephaly mapping 18](#_Toc535917161)

[SI 1.9: Major deviations from original proposed analysis plan 18](#_Toc535917162)

[Bibliography 18](#_Toc535917163)

# SI1.1: Arbovirus data description

The surveillance system and reporting procedures for notification of arboviral disease (SINAN) has already been described in detail in the context of the Zika outbreak previously [1]. Laboratory testing of notified arboviral databases is detailed in a separate database: GAL. Since Zika became a notifiable disease in December 2015, all suspected arboviral cases were referred for PCR testing in reference laboratories. This created a significant backlog in samples to be tested and hampered initial interpretation of confirmed Zika cases. Since then, retrospective testing has enabled better interpretation of these rates. In this section, we describe the spatiotemporal dynamics of suspected and PCR confirmed Zika, chikungunya and dengue cases in Brazil.

## SI1.1.1 Variation over time of suspected and confirmed cases

Before December 2015, reference laboratory testing for arbovirus cases was rare and sporadic, hence our choice to focus our analysis on data prior to this date. Suspected and confirmed cases follow similar temporal dynamics for all three diseases with, perhaps, the slight exception of a lower second peak in 2017 Zika cases among PCR confirmed cases.

## SI1.1.2. Variation over space of suspected cases

## SI1.1.3. Distribution of total cases at the municipality level

Our analysis relies on brief focal transmission of arboviral pathogens as this allows us to define a highly specific window and magnitude of exposure to women at different points in pregnancy. In the figures below we show that the median size of a Zika outbreak is around 8 suspected cases and only lasts for 3 weeks. This allows us to generate highly specific estimates of the timing of exposure to Zika for pregnant women living in affected areas.

## SI1.1.4. Distribution of outbreak duration at the municipality level

## SI1.1.5. Testing and confirmation rates (total)

| Zika | | | Chikungunya | | | Dengue | | |
| --- | --- | --- | --- | --- | --- | --- | --- | --- |
| Suspected | PCR Tested | PCR Confirmed | Suspected | PCR Tested | PCR Confirmed | Suspected | PCR Tested | PCR Confirmed |
| 195,287 | 111,267 | 16,880 | 436,533 | 221,237 | 110,401 | 908,927 | 566,282 | 161,484 |

## SI1.1.6. PCR confirmation rates by state

To test the assumption of uniform PCR testing of suspected arbovirus cases across Brazil we examined the proportion of suspected cases that were PCR tested disaggregated by state for each disease. Mean and 95% confidence intervals are also plotted (in grey). For Zika, the state of Santa Caterina had a marginally significantly higher tested rate (507 suspected cases, of which 418 were tested and 20 were positive) and Piaui had a marginally significantly lower testing rate (6775 suspected cases, of which 1339 were tested and 14 were confirmed). Because these states represent such a small proportion of Zika cases in our dataset (0.2%), we do not believe that they bias the results and that the assumption of universal availability of tested among reference laboratory samples holds. Similar marginal outliers were found for dengue and chikungunya testing rates.

## SI1.1.7. Testing fatigue analysis

Finally, we sought to test the hypothesis that testing rates decline over time. This is due to suggestions that once Zika has been detected in a particular municipality, clinical diagnosis is sufficient for case ascertainment and future cases are not tested. To test this hypothesis we fit a series of linear regressions in each municipality with the response variable “proportion of suspected cases that are PCR tested” and “week” as the covariate. This was only fit to data after the first case of Zika was detected in each municipality. Significance of the slope coefficient was evaluated using an F-test statistic.

Among 5,505 municipalities, only 152 had any statistical evidence of declining testing rates after first identification of Zika (p < 0.05). Among these, the median magnitude of this decline was 0.009 per week. Given the median duration of the Zika outbreak in each municipality was only 2 weeks (SI1.2.4), this equates to a less than 2% reduction in testing frequency between the start and end of the Zika outbreak. We therefore consider any decline due to testing fatigue negligible.

# SI1.2: Microcephaly data description

RESP is an emergency system put in place to report cases of congenital microcephaly. Between when the system was first deployed in 8 December 2015 and 27 May 2017 a total of 12,396 suspected microcephaly cases were notified. As of 27 May 2017, 2,906 (23%) of these have been confirmed, 6,244 (50%) discarded and 3,246 (26%) are still under investigation.

Cases are required to be notified as suspected microcephaly if the head circumference of the foetus at birth is less than or equal to 31.9cm for male or 31.5cm for female or if there are clear signs of cranial growth defects before, at or after birth. This threshold head circumference for suspected microcephaly has changed over time, with $\leq$33.0cm used prior to 8 December 2015 (before RESP was established) and $\leq$32.0cm used between 8 December 2015 and 14 March 2016. Following the final update, suspected cases notified between 8 December and 14 March to RESP were reviewed using the more stringent, up-to-date sex-specific definition. Cases that did not meet this post March 2016 definition were discarded to enable consistency in surveillance over time.

Among microcephaly with structural brain defects (MWSD) cases the majority (85%) were confirmed post partum but within 28 days of birth, with a smaller proportion (11%) not confirmed until after 28 days and only 4% confirmed in utero. 72% of confirmed cases received a TORCH(S) test with 95 and 71 positive for Syphilis and Toxoplasmosis respectively. Under 50% were tested for arbovirus infection and under 30% for Zika and given the limited window of detection of arboviral RNA and the limited pregnant women testing opportunities, this was not considered a reliable measure of presence or absence of exposure and not used in subsequent analyses.

The majority of microcephaly cases were confirmed by multiple methods with the most prominent being imaging techniques (used in 73% of confirmed cases) and laboratory testing (31%). Among the imaging techniques used, computerised tomography (CT) was most common (56%) followed by cranial ultrasound (41%) and magnetic resonance imaging (MRI) (8%). Only a small number of cases were confirmed using only clinical signs and epidemiological nexus (3%). Given the requirement to use specialist imaging techniques to confirm microcephaly, it might be expected that confirmation rates could vary geographically, and in particular be lower in the Northeast where healthcare expenditure is typically lower. To test this hypothesis, we plot the proportion of tested (i.e. confirmed or rejected) microcephaly cases below with mean and 95% confidence intervals in grey. This showed no clear regional differences in ability to test for microcephaly. We are, therefore, confident that analyses of MWSD cases are not biased by availability of diagnostic resources for confirmation.

Among MWSD cases, 262 deaths were reported with 101 occurring in utero giving a case fatality rate given live birth of 6%.

## SI1.2.1 Assessing selection bias by comparing the distribution of key characteristics between data sub-selections

Changes in the distribution of MWSD over time between different datasets


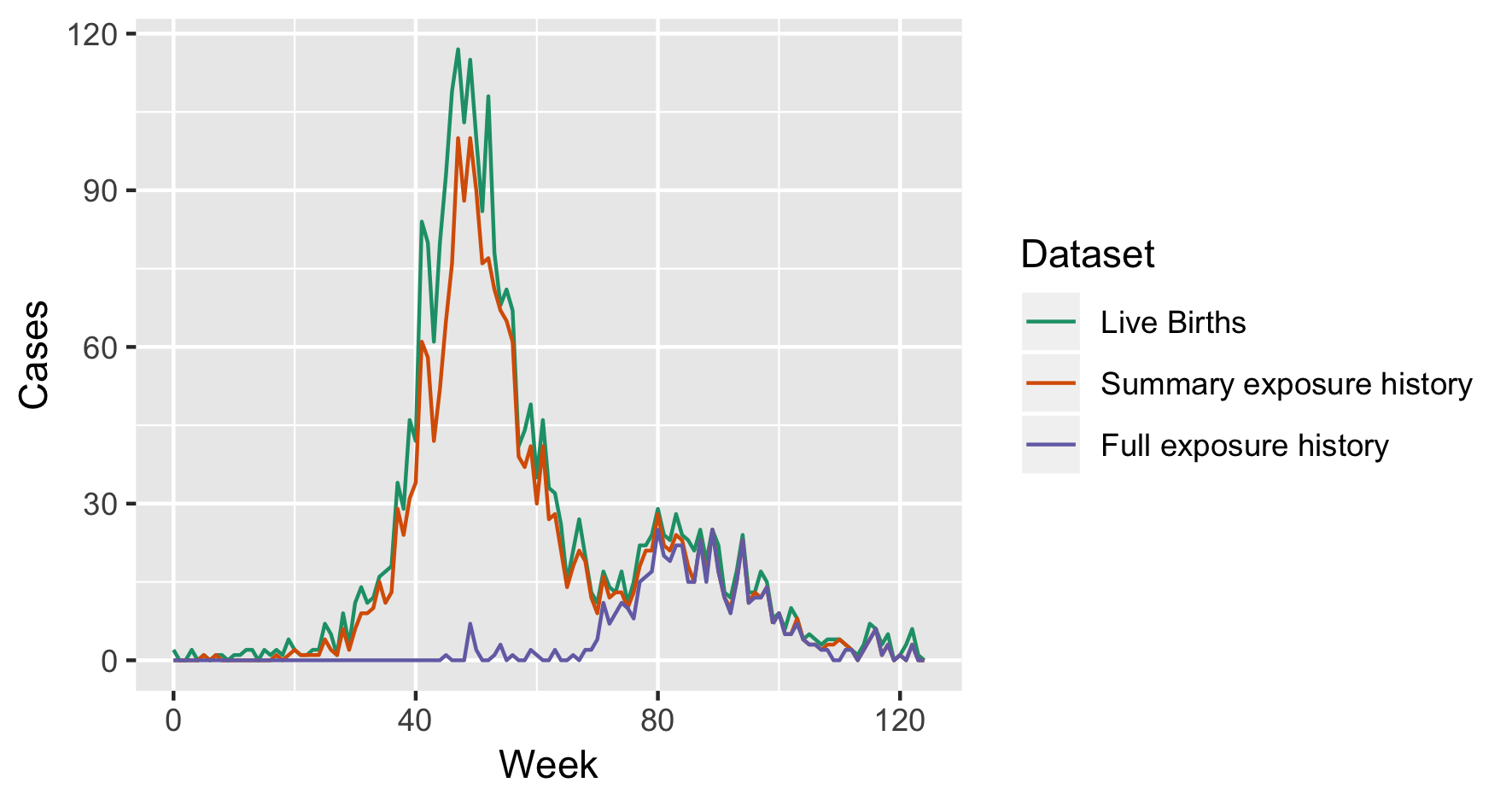


Spatial distribution (by state) of MWSD cases in different datasets


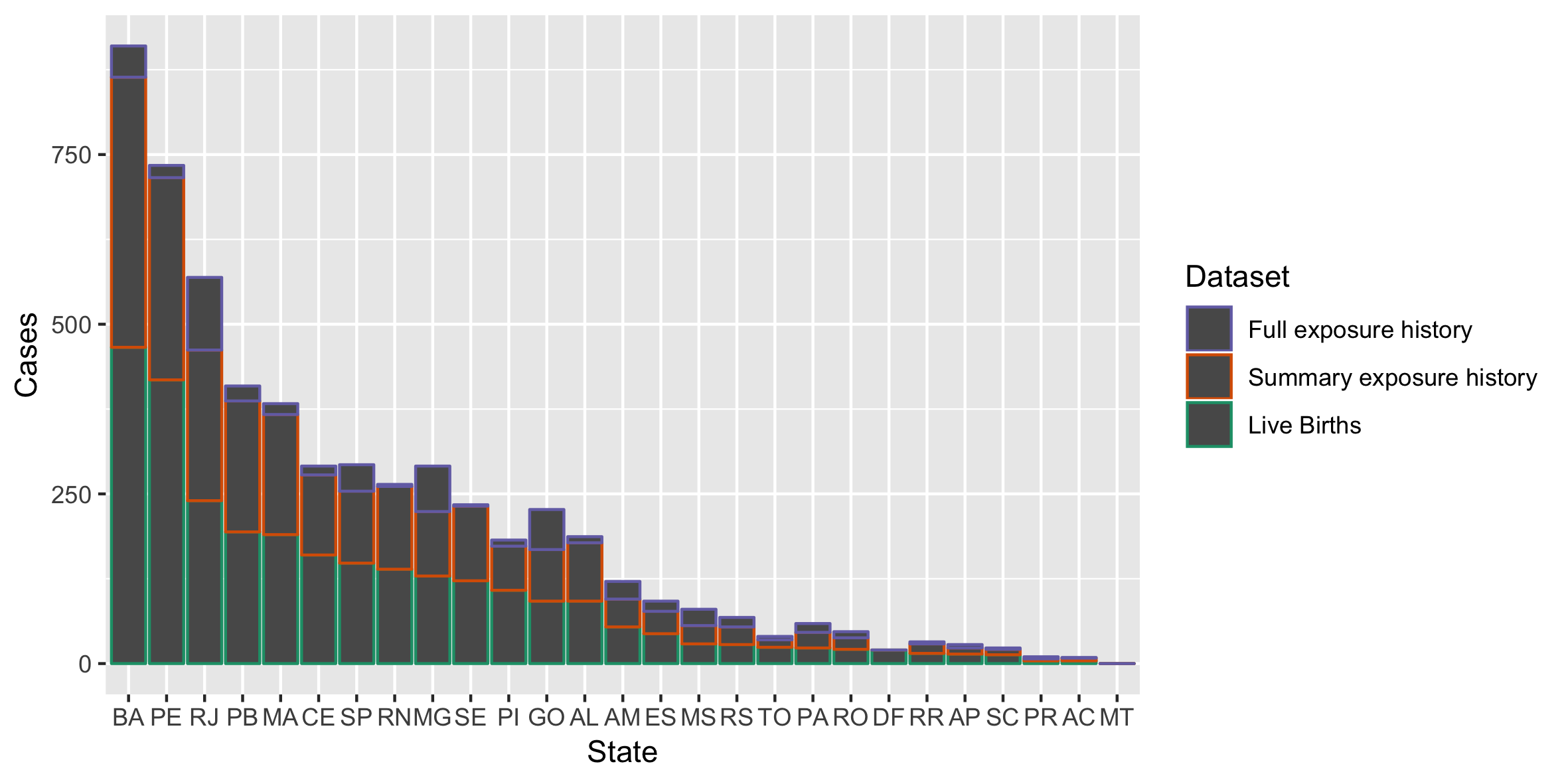


Age of mothers in different datasets


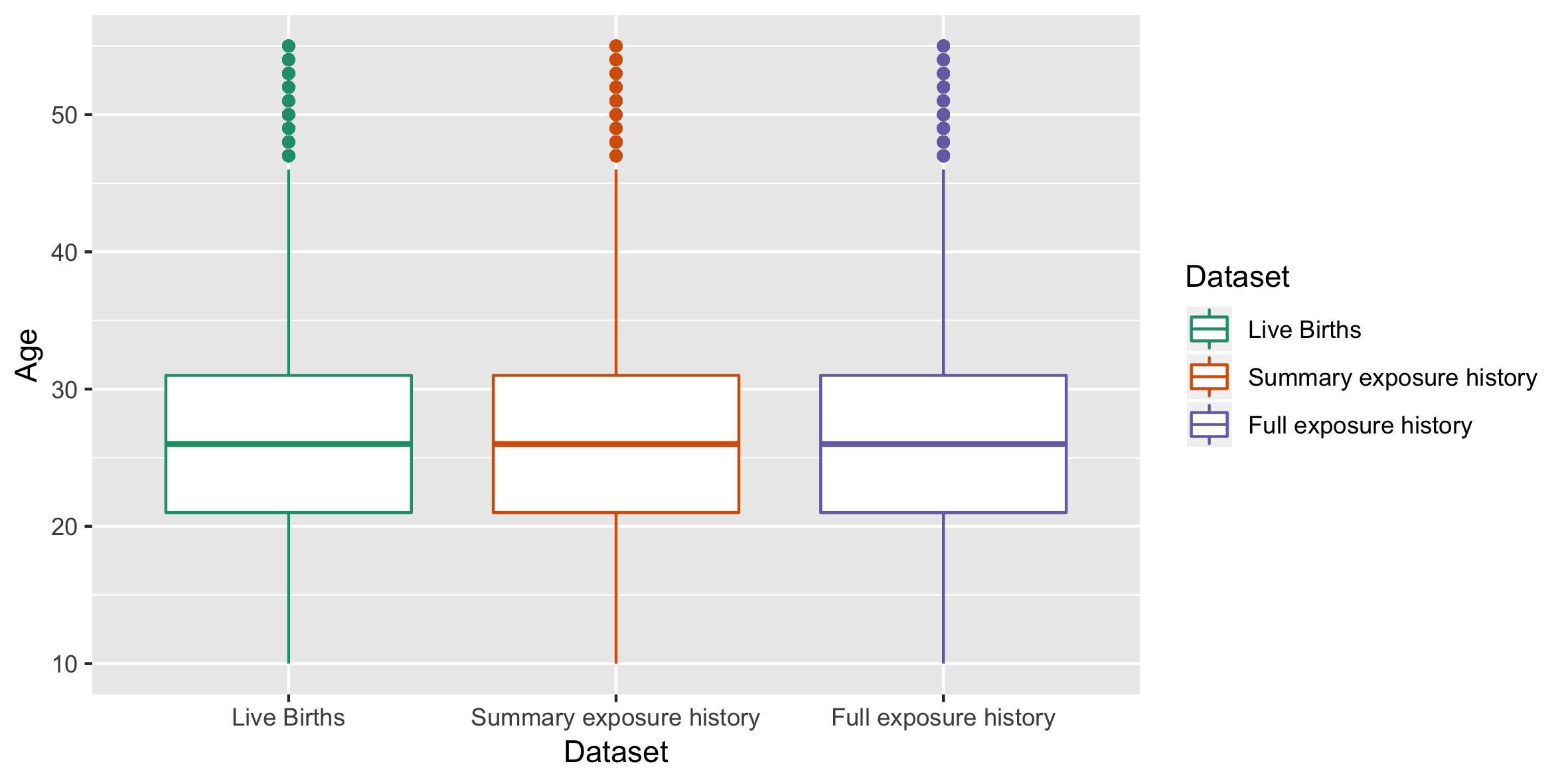


Proportion of male babies between different datasets


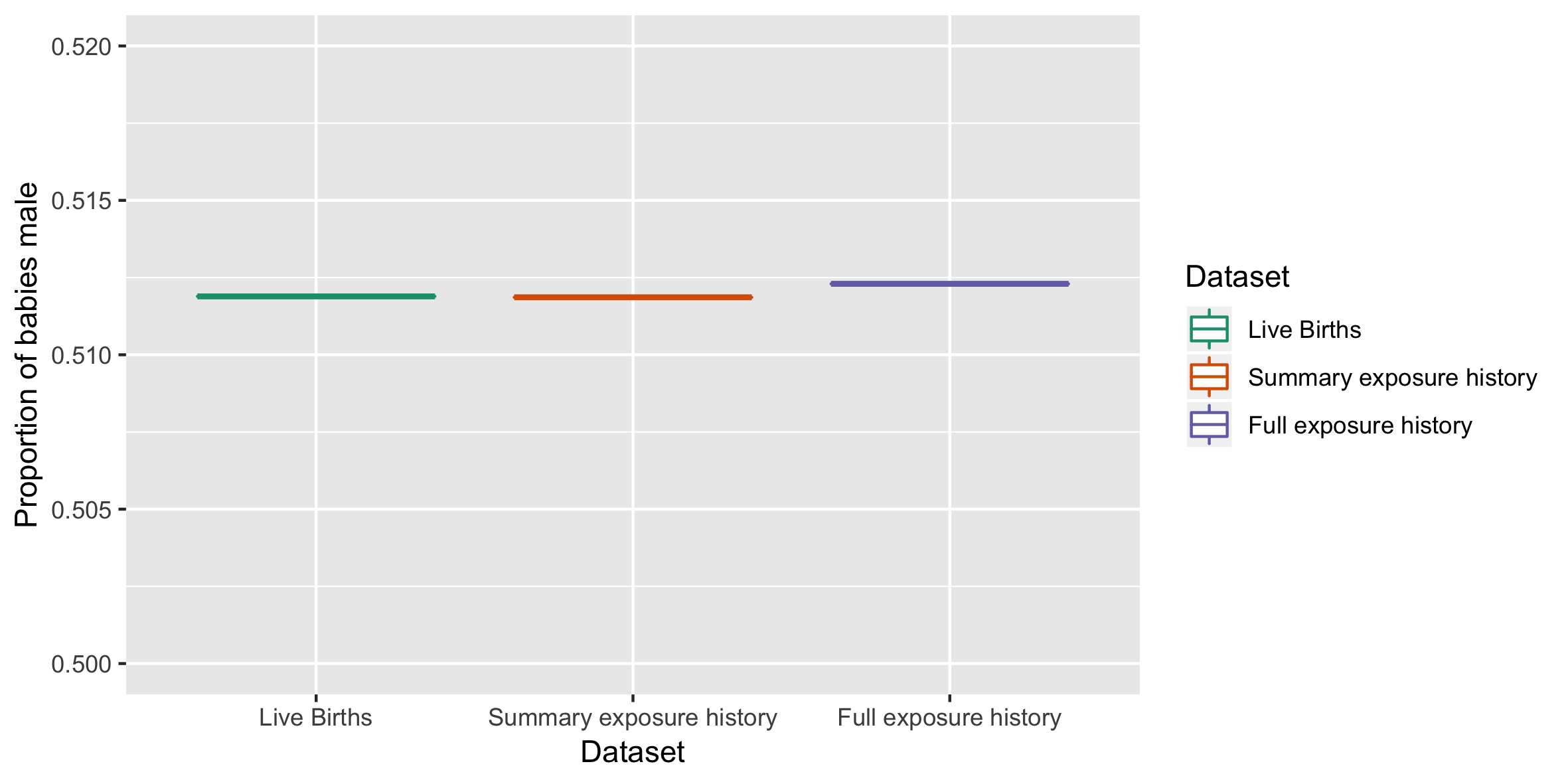


Socio-demographic Index differences between different datasets


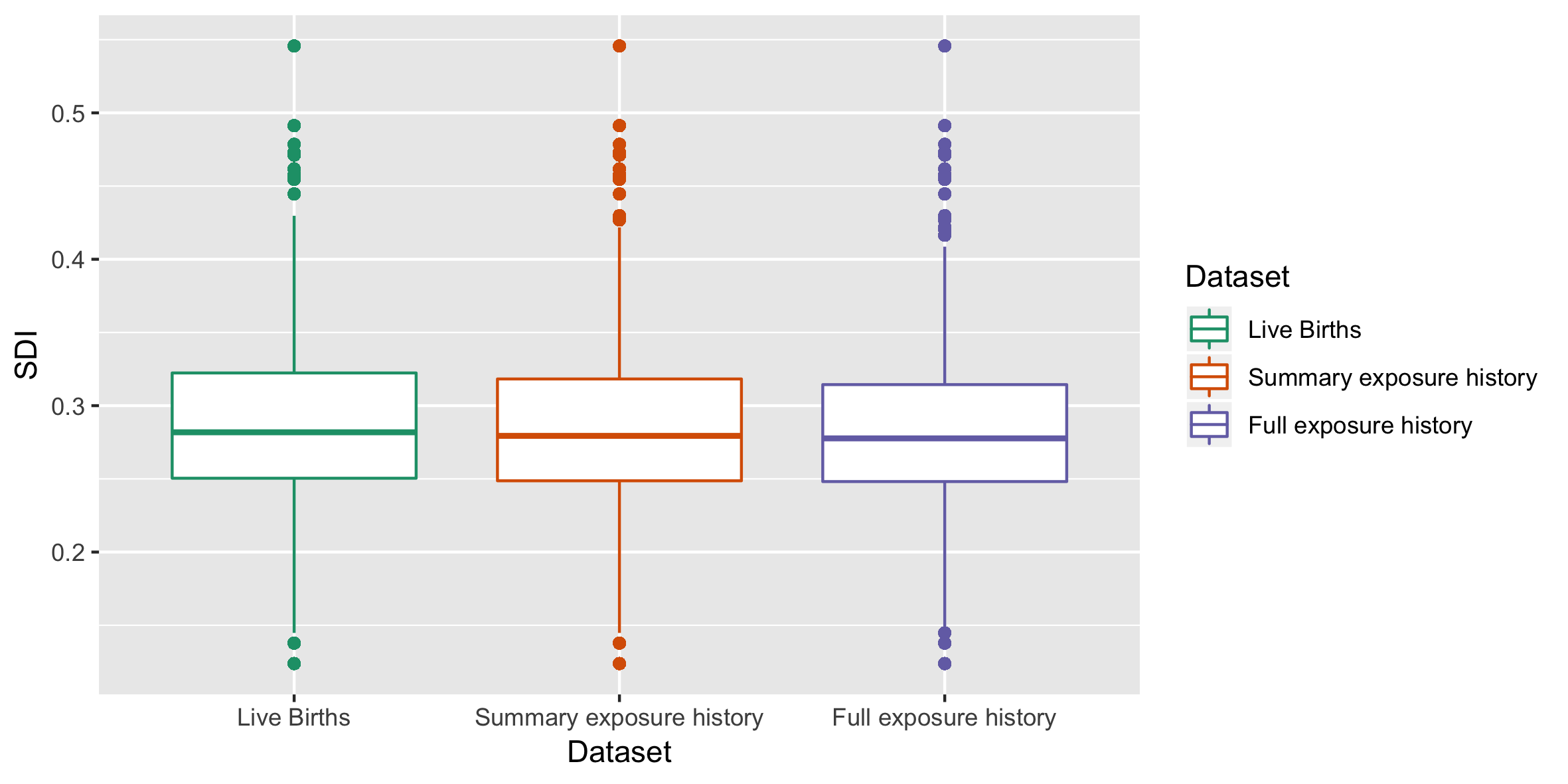


# SI1.3: Drinking water quality index construction

Access to safe drinking water is monitored continuously in Brazil through a series permits and surveys which are recorded in the SISAGUA national surveillance system [2]. On an annual basis, this system summarises the percentage of people within each municipality with access to three different water distribution mechanisms, including:

- Water supply systems (SAA): Continuous piped water infrastructure with direct delivery to the home residence.
- Collective alternative solution (SAC): Organised delivery of water to a communal source without the use of pipelines or permanent infrastructure
- Individual Alternative Solution (SAI): Individual water supply for personal domestic consumption (typically a self-dug well).

The system also records whether the water received treatment when delivered through SAA and SAC systems. Here we define a drinking water quality (DWQ) index for each municipality as the cumulative percentage of the population with access to untreated, self-obtained or water of unknown source:

DWQ = % untreated SAA + % untreated SAC + % SAI + % unknown source

This gave an approximate measure of relative risk of exposure to water-borne pathogens and its variation over space and time in Brazil. As data were not yet finalised for 2017, we assumed DWQ for the year 2017 was equal to DWQ in 2016.


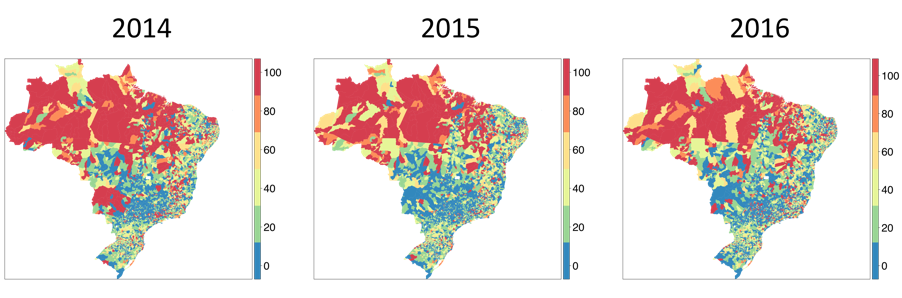


SI1.4 Missing data


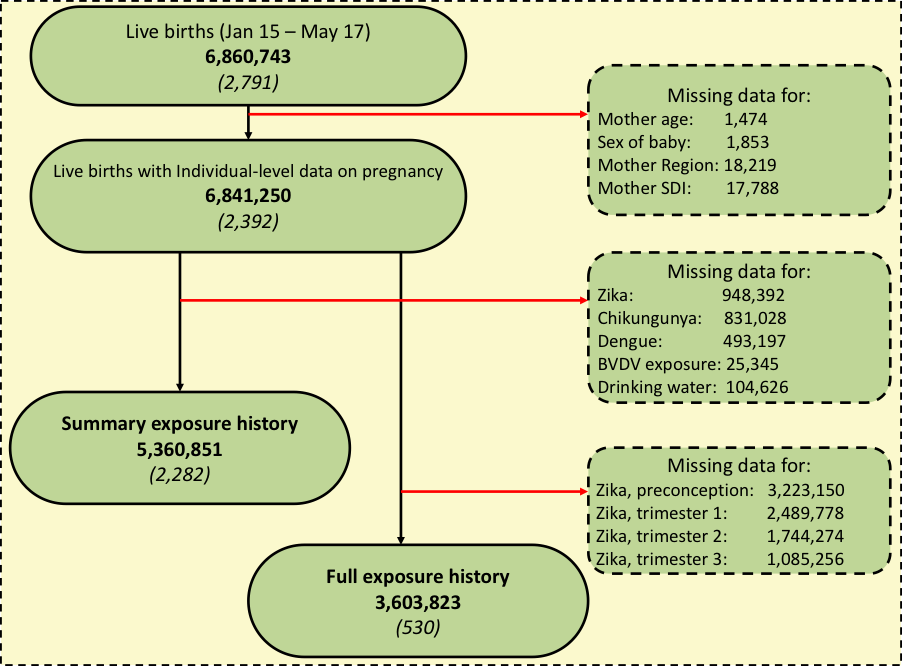


# SI1.5: Socio-demographic Index

The Socio-demographic Index aims to assess the relative development between municipalities, taking into account three key developmental metrics: income (mean dollars per capita), education (mean total years) and fertility (mean children per woman) collected during the 2010 Brazilian national census[3]. First minimum and maximum values of each metric are scaled between 0 (lowest municipality) and 1 (highest municipality). Then the arithmetic mean of the three scaled metrics for each municipality is used to create a relative Socio-demographic Index.

# SI1.6: Record linkage algorithm to match records in RESP to records in SINASC

The aim of this algorithm was to identify original birth records in the SINASC database that were subsequently updated with more contemporary or accurate information about microcephaly status of the child in the RESP database. We are therefore aiming to delete records from SINASC with good evidence of linkage with a record in RESP to avoid duplication.

Information fields common to both databases include: “date of birth”, “Sex of the baby”, “municipality of birth”, “state of birth”, “age of the mother”.

Due to the high completeness of these fields, we chose a simple matching algorithm that uses blocking by state, sex and age then finds the closest space-time match. The stages of the algorithm are available below and the R code files available in the linked GitHub repository [https://github.com/obrady/Brazil_microcepahly_analysis_public ].

To match individual record RESP[i]:

1: Subset SINASC records to the state of RESP[i]

2: Subset to same sex of child as RESP[i]

3: Subset to same age of mother (+/- 1 year) as RESP[i]

4: Compute distance (in meters) between the municipality midpoint of all records and the municipality midpoint of RESP[i]

5: Compute the absolute time distance (in days) of date of birth between all records and RESP[i]

6: Scale distance and time differences to fall within 0-1

7: Select and return the record that minimises the sum of time and space differences

The small number of records with missing data on state, sex of child and age of mother (SI1.4) was omitted from the relevant stage of the matching algorithm (i.e. included if missing).

When implemented, this algorithm performed as follows:

|  | Records identified in SINASC  (% of total number of RESP records) |
| --- | --- |
| Exact matches | 63% |
| Exact matches + within 30 days | 86% |
| Exact matches + within 30 days + close* municipalities | 96% |

* Close municipalities are those within 50 km

# SI1.7: Estimating the baseline rate of microcephaly

To estimate the risk of microcephaly due to non-Zika causes (the baseline rate) we made predictions using our final adjusted Zika model but with Zika exposure set to zero. Because data was missing on a small number of the model covariates multiple imputation was used to generate 100 datasets with missing data being replaced with random samples among the non missing values for each variable.

Within each imputed dataset, risk for each pregnancy in the absence of Zika was predicted using the fitted Zika model from the hypothesis analysis (Figure 2, main manuscript) then aggregated at municipality and national levels to generate rate predictions. Uncertainty in model coefficients (95% confidence intervals) was propagated to the next stage of the analysis.

To aggregate predictions across the 100 imputed datasets, 1,000 random samples were generated from a normal distribution parameterised by the mean and 95% CI predictions of baseline microcephaly rate (on a log scale). Combining the 100,000 samples build up a posterior distribution of the baseline microcephaly rate.


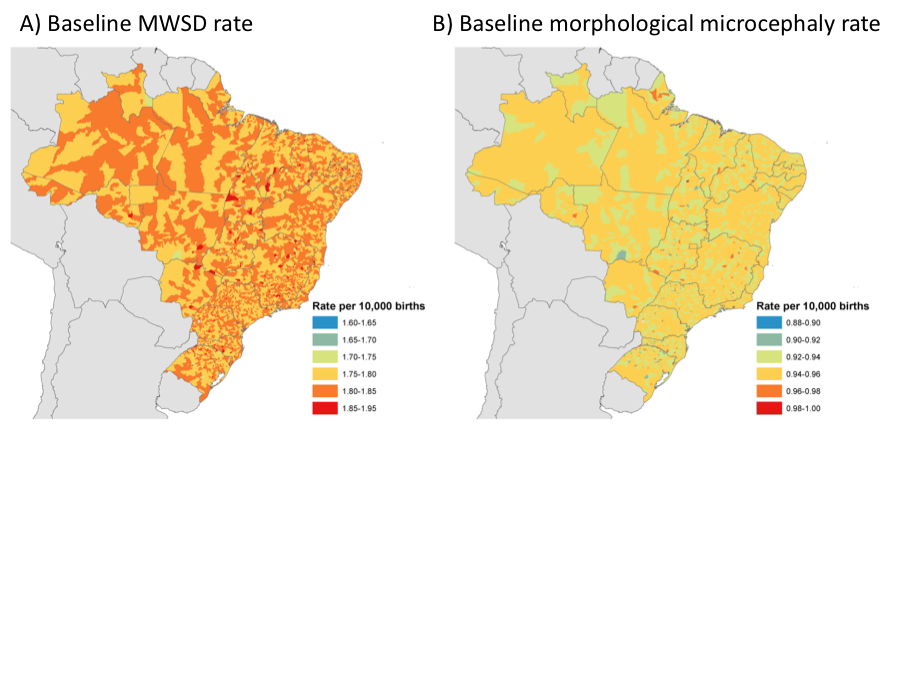


## SI1.8: Fit of GAM smoother to microcephaly data used in microcephaly mapping

**
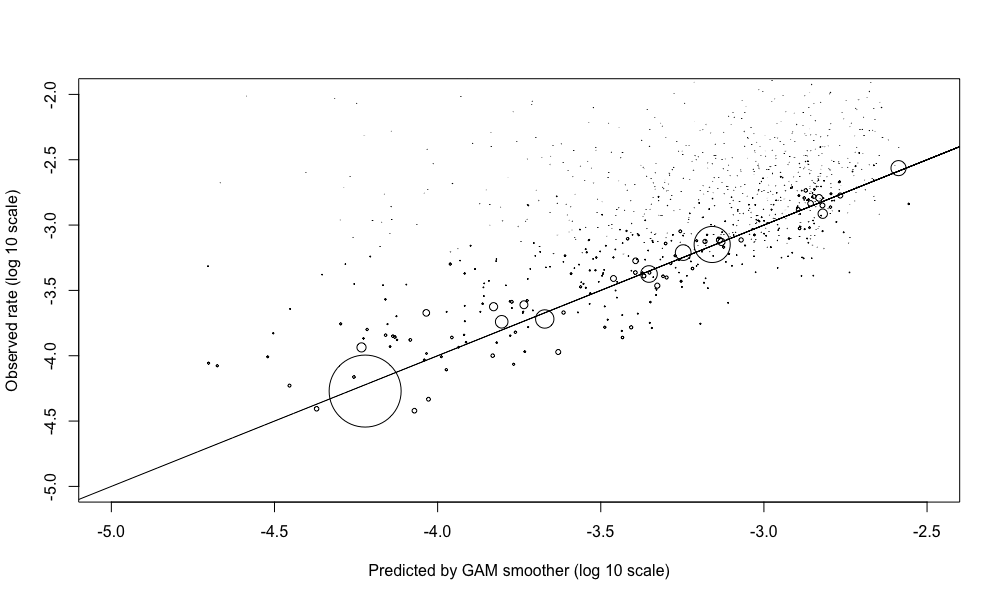
**

# SI 1.9: Major deviations from original proposed analysis plan

The majority of the analyses performed here were consistent with the prospective analysis plan (See supplementary information for original analysis plan). The biggest deviation was the selection of two separate datasets (summary and full exposure history) and their use for different analyses. As argued in the text, this was made necessary by the late establishment of ZIKV surveillance meaning that information on exposure was entirely missing or only partially complete for many pregnancies, particularly at the time when the majority of MWSD cases occurred. The implications of this change on the results in table 2 (estimated associations between candidate causes and MWSD) are shown below.


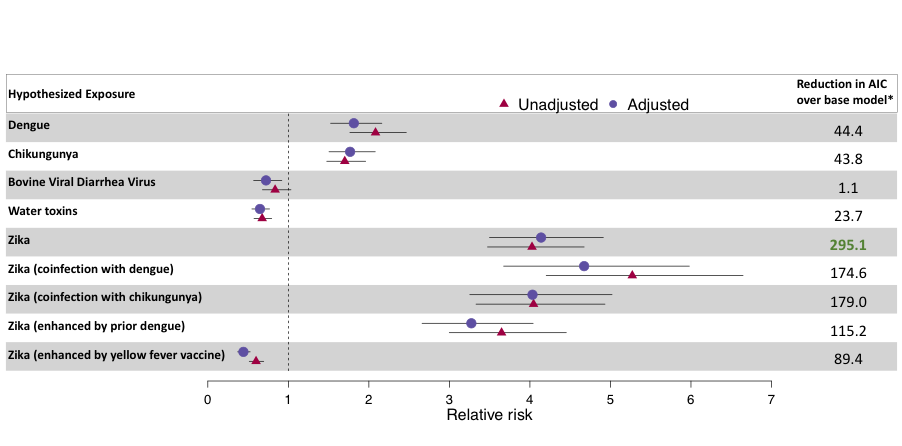


Figure 2 BEFORE the use of summary exposure datasets

Figure 2 AFTER the use of summary exposure datasets (as presented in the main manuscript)

Most importantly, this did not change our principle finding that Zika and Zika alone (i.e. no effect modifiers) is the most parsimonious explanation for the observed spike in MWSD cases 2015-2017 in Brazil as demonstrated by the biggest reduction in AIC over the base model. We did notice, however that the use of the summary exposure dataset does bias the estimate of relative risk towards the null. This is because the summary exposure dataset only has information on exposure late in pregnancy for many women and therefore differentially classifies them as unexposed when they may have been exposed earlier in pregnancy. This shaped our decision to derive our final stated estimates of relative and absolute risk using the full exposure dataset and time-specific exposure model (Figure 1 main manuscript) in the final analysis. We also noted that the use of the summary exposure dataset led to a protective rather than enhancing inference about the effects of dengue exposure in pregnancy. Neither model gave a particularly parsimonious fit with the data (low reduction in AIC over the base model when compared to the Zika-only model) and we therefore do not interpret this change as particularly meaningful.

We also failed to anticipate the need for a formal record linkage algorithm to formally link RESP and SINASC databases as we assumed that the presence of a RESP record would be indicated in a distinct field in SINASC which turned out to be unreliable.

# Bibliography

1. de Oliveira WK, de França GVA, Carmo EH, Duncan BB, de Souza Kuchenbecker R, Schmidt MI. Infection-related microcephaly after the 2015 and 2016 Zika virus outbreaks in Brazil: a surveillance-based analysis. Lancet. Elsevier; 2017;390: 861–870. doi:10.1016/S0140-6736(17)31368-5

2. Vasconcelos CH, Andrade RC de, Bonfim CV, Resende RM de S, Queiroz FB de, Daniel MHB, et al. Surveillance of the drinking water quality din the Legal Amazon: analysis of vulnerable areas. Cad Saúde Coletiva. Instituto de Estudos em Saúde Coletiva da Universidade Federal do Rio de Janeiro; 2016;24: 14–20. doi:10.1590/1414-462X201500040142

3. Instituto Brasileiro de Geografia e Estatística. IBGE : 2010 Population census results [Internet]. [cited 23 Feb 2018]. Available: https://ww2.ibge.gov.br/english/estatistica/populacao/censo2010/default.shtm
