# Supplementary material for: The association between Zika virus infection and microcephaly in Brazil 2015–2017: An observational analysis of over 4 million births
Source: PLoS Med. 2019 Mar 5;16(3):e1002755. doi: 10.1371/journal.pmed.1002755 (PMC6400331; doi:10.1371/journal.pmed.1002755)
Supplement: S2 Text — (DOCX) [file pmed.1002755.s005.docx]

S2 Text to:

“The association between Zika and microcephaly: an observational analysis of over four million births in Brazil 2015-2017”

Table of Contents

[SI2.0: Testing the influence of normal infant size on the Zika microcephaly association 2](#_Toc535916958)

[SI2.1: Measuring change in birth rate over the course of the Zika outbreak 3](#_Toc535916959)

[SI2.2: Examining the association between Zika and microcephaly using an alternative municipality-level Poisson model structure 5](#_Toc535916960)

[SI2.3: Sub-analysis to test for the involvement of Race in the Zika-microcephaly association 6](#_Toc535916961)

[SI2.4: Results when using suspected arbovirus and microcephaly cases 6](#_Toc535916962)

[SI2.5: Assessing the influence of collinearity of Zika exposure in the first two trimesters of pregnancy 10](#_Toc535916963)

[SI2.6: full ICD-10 codes for congenital abnormality categories 11](#_Toc535916964)

[SI2.7: The rate of reported congenital abnormalities by category over time. 12](#_Toc535916965)

[SI2.8: Examining Zika model residuals 12](#_Toc535916966)

[SI2.9: Covariates included in the final adjusted models 13](#_Toc535916967)

# SI2.0: Testing the influence of normal infant size on the Zika microcephaly association

One alternative hypothesis why rates of microcephaly were higher in the northeast is that infants are typically smaller at birth in the northeast region (due to factors such as lower maternal nutrition standards) compared to the national average, meaning that the national definition for microcephaly based on head circumference may be more sensitive and less specific in this region.

While we have already tried to minimize and influence of this in our analysis by using imaging confirmed (structural) microcephaly cases, rather than just the suspected cases, to formally test this hypothesis, municipality level mean Z score for weight was calculated using all healthy births (i.e. those without notified birth defects in the SINASC database). Head circumference or infant length was not available for all births, so weight was used as a general proxy of infant size. The most likely model from the logistic regression analysis (Zika exposure) was then compared to the same model with the addition of the Z score in the area of residence of the mother and to a model where Zika was omitted and only the Z score in the local area was included. If the association between Zika and microcephaly is confounded by average birth size then municipality Z score should either provide the most parsimonious model (biggest reduction in AIC), or at least increase the parsimony of the Zika model when included as a covariate.

The results are summarised in the below table that shows that variation in the size of healthy infants at birth does not explain the observed pattern of microcephaly (higher AIC than base model). It does provide a marginal increase in parsimony when included as an additional covariate with Zika as the main exposure (AIC reduction of 297 vs 295, chi-squared = 0.0279). This might suggest that lower than average birthweight in some areas may have a small confounding effect in the association between congenital ZIKV infection and microcephaly, likely through increased diagnostic sensitivity. However, given the marginal improvements in model parsimony, a p-value for mode difference of > 0.01 and reduced interpretability of the mode with this covariate, we chose not to include the covariate average weight Z-score in municipality of residence of the mother in further analyses.

**Comparison of Zika only, Zika + local Z score and local Z score only models**

* All models included the stated covariates plus the basic variables of week, region, mother age and sex of the baby to account for the variable background rate of microcephaly. ** the base model contains covariates for week, region, mother age and sex of the baby.

| Model covariates* | Reduction in AIC over base model** |
| --- | --- |
| Zika | 301.35 |
| Zika, mean local Z score | 301.83 |
| Mean local Z score | 22.91 |

# SI2.1: Measuring change in birth rate over the course of the Zika outbreak

Since the declaration of Zika as a public health emergency and the wide publicizing of Zika’s link with congenital abnormalities, it has been hypothesised that women would avoid or delay pregnancy or possibly attempt to have (illegal) abortions. Figure SI2 shows the normalised weekly number of births in the five regions of Brazil and national totals. This shows that there was a highly significant (all p < 0.001, two-sided t-test) reduction in the number of births in late 2016- early 2017 in coinciding with an approximate 40 week lag from the declaration of Zika as a public health emergency.

Importantly, this reduction happened at the same time in all regions despite the Zika outbreak affecting these regions at different times. This suggests that the decision to delay or defer pregnancy was a synchronised mass reaction to Zika’s international status, not a response specific to local ZIKV dynamics. This reduces the bias imposed by such a change because it affects pregnant women both with and without the exposure (i.e. it is non-differential).

Over this period, these regions experienced an approximate 15% reduction in the number of births (1.5 million fewer births), with the Centralwest region worst affected (32.7% reduction). All regions did, however, see a subsequent rapid increase in the number of births in early 2017, a period when the birth rate is typically stable or in decline. This is consistent with temporary delay of pregnancy in a minority of the population and is less consistent with abortion or a permanent reduction in fertility. As our analysis is conducted at an individual birth level, this temporary universal (non-differential) reduction in birth rate will not significantly affect the findings of any of these analyses.

Only when further data becomes available for later time periods in 2017 will it be possible to determine if this temporary reduction in the number of births will be compensated for with higher birth rates later in 2017.

**Weekly birth rate in Brazilian regions over the course of the Zika epidemic.**

Births are plotted on a median normalised scale to allow comparisons between regions. Black points indicate data from the dates 1^st^ January 2015 – 1^st^ September 2016 (40 weeks after the declaration of Zika as a public health emergency) and red points show data after this time period. A sinusoidal trend line ($\pm$ two standard error of the fit) fit to the black data points is added to show regular seasonal variation. Residuals between the predicted trend line and observed birth rates (red dots) were highly significant (two sided t-test, p < 0.001 for all provinces).

**Average percentage deviation in birth rate 01/09/2016 – 16/03/17**

| **Area** | **Mean percentage reduction from predicted birth rate** |
| --- | --- |
| Centralwest | 32.7 |
| North | 6.1 |
| Northeast | 13.8 |
| South | 12.1 |
| Southeast | 14.8 |
| **National** | **14.7** |

# SI2.2: Examining the association between Zika and microcephaly using an alternative municipality-level Poisson model structure

Because many of the covariates used in this analysis have been estimated using the timing and location of residence of each mother, we performed a sensitivity analysis to our estimation of Zika exposure method. To do this we performed a simplified version of the analysis where only information aggregated at the municipality level and over the entire time period of the study was used. We were, therefore testing the hypothesis that cumulative Zika incidence in an area over the three-year study period is associated with higher microcephaly rate and the relative likelihood of this association in comparison with other candidate exposures.

As in the main analysis, confounders for Region of the municipality, mean mother age in the municipality, and socio-demographic index of the municipality were included and backwards selected by AIC in a Poisson generalised linear model. Model relative likelihood was assessed using reduction in AIC from the base model which just contained the above selected confounders and no exposure variables. The results of this analysis are shown in the below table. Using this alternative more simplistic dataset and model structure does not alter the conclusion that Zika alone is the most likely cause of the elevated rates of microcephaly observed in Northeast Brazil.

| Candidate exposure | Reduction in AIC over the base model |
| --- | --- |
| Dengue | -0.01 |
| Chikungunya | 17.4 |
| BVDV | 7.65 |
| Water toxins | 106.44 |
| Zika | 228.28 |
| Zika and dengue | 26.27 |
| Zika and chikungunya | 89.98 |
| Zika and Yellow fever vaccine | 112.58 |

# SI2.3: Sub-analysis to test for the involvement of Race in the Zika-microcephaly association

To test if race of the mother affected the association between Zika and microcephaly a further set of analyses was undertaken. Because information on race of the mother was missing from further pregnancy records, we had to further trim the “All exposures” dataset down to 3,843,891 pregnancies and 443 cases of microcephaly with structural brain defects (MWSD). Race was categorised as follows:

| Race category | Total |
| --- | --- |
| Amarela (“Yellow”) | 14,741 |
| Branca (“White”) | 1,428,835 |
| Indigena (“Indigenous”) | 23,471 |
| Negra (“Black”) | 213,707 |
| Parda (“Brown”) | 2,163,137 |

We tested the inclusion of mother race in three different ways: i) as a confounder that acts independently of Zika, ii) as an effect modifier of Zika, and iii) as the sole determinant of microcephaly variation in the absence of Zika. All models also included covariates for mother age, sex of the baby, time and region.

| Main exposure | Crude Model AIC |
| --- | --- |
| Zika | -333.08 |
| Zika + mother race | -328.97 |
| Zika : mother race | -327.59 |
| Mother race | -81.36 |

: denotes an interaction term

This showed that and addition of a mother race variable increased model AIC and therefore led to a less parsimonious model. Furthermore none of the individual race terms were significant at the p = 0.05 level. We therefore conclude that mother race is not a significant factor influencing the baseline rate of microcephaly or the Zika-microcephaly association.

# SI2.4: Results when using suspected arbovirus and microcephaly cases

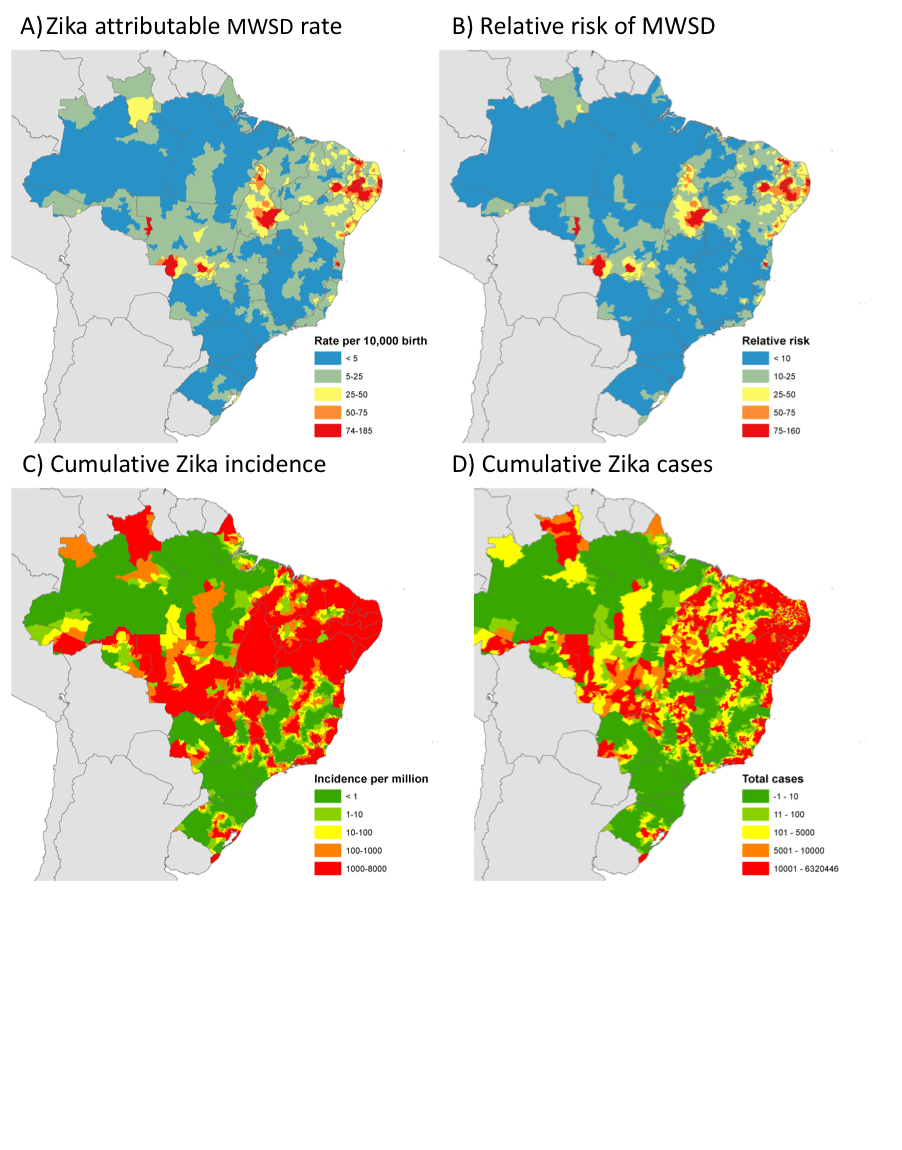

| Region | Estimated cases | Percentage of burden | Incidence per 100,000 residents | Percentage of cases reported |
| --- | --- | --- | --- | --- |
| Central-West | **5,646,557**  (4,410,559-6,685,693) | **6.82**  (6.02-7.27) | **40,248.3**  (31,438.2-47,655.2) | **0.33**  (0.28-0.43) |
| North | **3,523,463**  (3,257,515-3,834,653) | **4.26**  (4.17-4.44) | **24,361.9**  (22,523.1-26,513.6) | **0.92**  (0.84-0.99) |
| North-East | **44,102,729**  (41,682,074-45,874,123) | **53.30**  (49.91-56.86) | **84,263.0**  (79,638.1-87,647.5) | **0.13**  (0.12-0.13) |
| South | **2,187,061**  (1,814,510-2,695,760) | **2.64**  (2.48-2.93) | **8,008.2**  (6,644.1-9,870.9) | **0.38**  (0.31-0.46) |
| South-East | **27,290,796**  (22,141,491-32,831,214) | **32.98**  (30.20-35.72) | **34,780.3**  (28,217.9-41,841.2) | **0.29**  (0.24-0.36) |
| Total | **82,750,607**  (73,306,151-91,921,443) | **100** | **44,344.6**  (39,283.5-49,259.1) | **0.24**  (0.21-0.27) |

# SI2.5: Assessing the influence of collinearity of Zika exposure in the first two trimesters of pregnancy

Cumulative Zika incidence in Trimesters 1 and 2 were found to be colinear (R^2^ = 0.75) so were combined in the main paper analysis. Further models were fit to investigate the possible consequences of this combination. First, both variables (T1 and T2) were included as if they were not colinear (the “full model” in the below figure) and second, two models were fitted in which either variable was omitted. The predicted coefficient for T1 and T2 from each of these three models are shown in the below figure. In both the full model and when comparing the two exclusion models, exposure to Zika in the second trimester of pregnancy appear to confer a higher risk of microcephaly at birth. Combination of exposure data into a combined T1-2 variable may bias the relative risk estimates for this exposure towards the null and underestimate total risk, but given the high collinearity between T1 and T2, combination provides the most statistically robust inference from this dataset.

**Estimated coefficient of Zika exposure at different trimesters of pregnancy in the microcephaly model**. T1 = trimester 1, T2 = trimester 2. All models also included variables for sex of the baby, time and location variables (see main manuscript) , ZIKV exposure in the 10 weeks prior to conception and ZIKV exposure in Trimester 3.


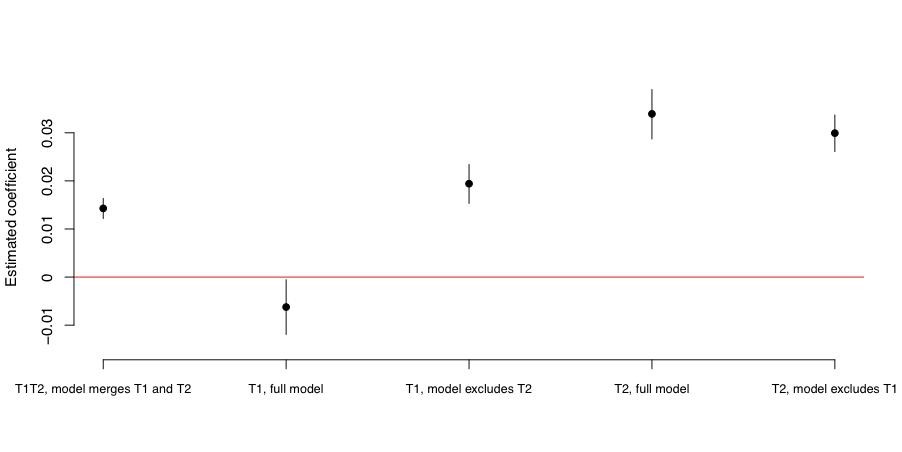


# SI2.6: full ICD-10 codes for congenital abnormality categories

| **Defect categories** | **ICD-10 codes** |
| --- | --- |
| All non-microcephaly notified birth defects | Q00-Q99 excluding Q0.20 (microcephaly) |
| Brain and Central nervous system | Q0.00 - Q0.70 excluding Q0.20 (microcephaly) |
| Eye | Congenital malformations of eye  Q10.0 - Q15.0 |
| Musculoskeletal including limb abnormalities | Congenital malformations and deformations of the musculoskeletal system  Q65.0 - Q79.0 |
| Head and neck including cleft lip and cleft palate | Congenital malformations of ear, face and neck  Q16.0 - Q18.0  Congenital malformations of the nose  Q30.0-Q30.9  Cleft lip and cleft palate  Q35.0 - Q37.0 |
| Cleft palate | Q359 |
| Down Syndrome | Q909 |
| Hydrocephalus | Q039 |
| Gastroschisis | Q793 |
| Feet deformities | Q668 |
| Hypospadias | Q549 |
| Polydactyly | Q690 and Q699 |

# SI2.7: The rate of reported congenital abnormalities by category over time.

# SI2.8: Examining Zika model residuals

The residuals (observed MWSD minus predicted MWSD) of the time-specific exposure model from which we derive our best estimates of ZIKV relative and aboluse risk were examined with respect to space and time to assess overall model fit.

Upper: model residuals of microcephaly with structural brain defects (MWSD) over time (weeks since 01 January 2015). Lower: Spatial distribution of model residuals by state in Brazil.

# SI2.9: Covariates included in the final adjusted models

**Hypothesis testing models (Figure 2)**

| **Hypothesised exposure** | **Main effect** | **Retained covariates after model selection*** |
| --- | --- | --- |
| Baseline model | - | Region |
| Dengue | Dengue incidence^1^ | Region |
| Chikungunya | Chikungunya incidence^1^ | Region |
| Bovine Viral Diarrhea Virus | Farm density | Sex of baby  Region |
| Water Toxins | Water vulnerability index | Region |
| Zika | Zika incidence^1^ | Sex of baby  Region |
| Zika (coinfection with dengue) | (Zika incidence^1^):(Dengue incidence^1^) | Region |
| Zika (coinfection with chikungunya) | (Zika incidence^1^):(Chikungunya incidence^1^) | Sex of baby  Region |
| Zika (enhanced by yellow fever vaccination) | (Zika incidence^1^):(Yellow fever vaccination coverage^2^) | Region |

* covariate options include: Age of mother, Sex of baby, Week, Region, and

Socio-demographic Index (SDI) level

^1^ Arbovirus incidence refers to mean incidence in the municipality of residence of the mother over the course of her pregnancy

^2^ Yellow fever vaccination coverage in the area of residence of the mother in the year in which the pregnancy began

The symbol “:” denotes an interaction between two variables (product terms)

**Other birth defect outcomes (Figure 5)**

| **Birth defect** | **Main effect** | **Retained covariates after model selection*** |
| --- | --- | --- |
| All non-microcephaly | Zika incidence^1^ | Week |
| All brain | Zika incidence^1^ | Week  Region  SDI |
| All eye | Zika incidence^1^ | Sex of baby  Region |
| All musculoskeletal | Zika incidence^1^ | Week  SDI |
| All head and neck | Zika incidence^1^ | Week |
| Cleft palate | Zika incidence^1^ | Age of mother |
| Down syndrome | Zika incidence^1^ | Week |
| Hydrocephalus | Zika incidence^1^ | Age of mother  Region  SDI |
| Gastroschisis | Zika incidence^1^ | Region |
| Feet deformities | Zika incidence^1^ | Age of mother |
| Hypospadias | Zika incidence^1^ | Week  SDI |
| Polydactyly | Zika incidence^1^ | Sex of baby |

* covariate options include: Age of mother, Sex of baby, Week, Region,

and Socio-demographic Index (SDI) level

^1^ Arbovirus incidence refers to mean incidence in the municipality of residence of the mother over the course of her pregnancy
